# Recent advances in genetic systems in obligate intracellular human-pathogenic bacteria

**DOI:** 10.3389/fcimb.2023.1202245

**Published:** 2023-06-19

**Authors:** Derek J. Fisher, Paul A. Beare

**Affiliations:** ^1^ School of Biological Sciences, Southern Illinois University, Carbondale, IL, United States; ^2^ Rocky Mountain Laboratory, National Institute of Health, Hamilton, MT, United States

**Keywords:** *Chlamydia*, *Coxiella*, *Rickettsia*, *Anaplasma*, *Ehrlichia*, *Orientia*, genetics, obligate

## Abstract

The ability to genetically manipulate a pathogen is fundamental to discovering factors governing host–pathogen interactions at the molecular level and is critical for devising treatment and prevention strategies. While the genetic “toolbox” for many important bacterial pathogens is extensive, approaches for modifying obligate intracellular bacterial pathogens were classically limited due in part to the uniqueness of their obligatory lifestyles. Many researchers have confronted these challenges over the past two and a half decades leading to the development of multiple approaches to construct plasmid-bearing recombinant strains and chromosomal gene inactivation and deletion mutants, along with gene-silencing methods enabling the study of essential genes. This review will highlight seminal genetic achievements and recent developments (past 5 years) for *Anaplasma* spp., *Rickettsia* spp., *Chlamydia* spp., and *Coxiella burnetii* including progress being made for the still intractable *Orientia tsutsugamushi*. Alongside commentary of the strengths and weaknesses of the various approaches, future research directions will be discussed to include methods for *C*. *burnetii* that should have utility in the other obligate intracellular bacteria. Collectively, the future appears bright for unraveling the molecular pathogenic mechanisms of these significant pathogens.

## Introduction

Obligate intracellular bacterial pathogens by their unique nature have an intimate relationship with the human host. Residence within their respective eukaryotic cellular niches provides opportunities to parasitize the host cell for nutrients, driving a reductive evolutionary process resulting in the significantly reduced genomes these bacteria exhibit compared with most free-living bacteria. An intracellular lifestyle also presents unique immunological challenges for the pathogen such as the need to avoid host-level and cellular-level immune responses while maintaining host cell viability long enough to support replication, allowing for pathogen expansion and transmission. The dynamic interactions between the host cell and its bacterial endosymbiont are mediated by an intricate interplay of both host and bacterial proteins, signaling molecules, metabolites, etc., all of which owe their existence to genetic adaptation. Dissecting these relationships at the molecular level is essential for understanding host–pathogen dynamics and, ultimately, the development of preventative measures and therapeutic approaches.

The most powerful method for elucidating the importance of a specific trait to disease, whether it be a host- or pathogen-encoded trait, is to construct a mutant strain via gene inactivation, altered gene expression levels, or introduction of a gene into an organism providing a gain of function. The vast majority of the methods used for these approaches in bacteria requires introduction of nucleic acid into the pathogen of interest. In this regard, obligate intracellular pathogens have largely taken a backseat to extracellular and facultative intracellular pathogens for a myriad of reasons that will be enumerated in the coming sections. Therefore, while genetic manipulation via transformation of *Escherichia coli* has been feasible since 1970 (Mandel and Higa, 1970), stable transformation of obligate intracellular pathogens was not achieved until 1996 with *Coxiella burnetii* (Suhan et al., 1996) followed by *Rickettsia* spp. in 1998 (Rachek et al., 1998) and *Chlamydia trachomatis* in 2011 (Wang et al., 2011). Genetic systems for *Ehrlichia* and *Anaplasma* have been in use for ~15 years (Long et al., 2005; Felsheim et al., 2006) but remain limited in number, and while successes have been achieved for obligate intracellular bacteria, important human pathogens including *Orientia tsutsugamushi* and *Mycobacterium leprae*, the animal pathogen *Lawsonia intracellularis*, and numerous insect endosymbionts remain genetically intractable. The inability to readily manipulate the genomes of obligate intracellular bacteria has been a significant barrier to research and creates bottlenecks in our lines of inquiry due to simple technical limitations. For example, fulfillment of Molecular Koch’s postulates (Falkow, 1988), a classic approach for determining the role of a gene in pathogenesis whereby a gene inactivated mutant is studied in parallel to a strain with the gene restored via complementation, has only recently been attainable for most of the pathogens covered in this review.

We will summarize general considerations for genetic systems in obligate intracellular human–pathogenic bacteria, discuss the current state-of-the-art methods employed by researchers, and provide our perspectives on future research directions. While a holistic overview of genetic systems will be provided, primary focus will be given to achievements since 2017 and we refer readers to the excellent review by McClure et al. for details on prior successes (McClure et al., 2017).

## General considerations when developing genetic systems

### Organism lifestyle, culturing conditions, and developmental forms

**Figure 1:**
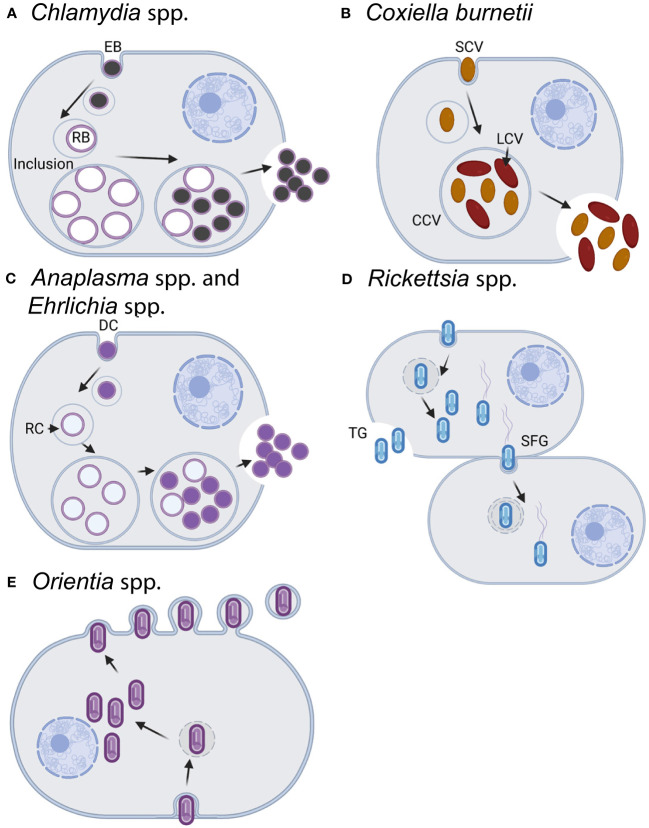
Infection and replication lifestyles of select obligate intracellular bacteria in the animal host. **(A)** *Chlamydia* spp. undergo a biphasic development cycle with the infectious elementary body (EB) internalized into the host cell (primarily epithelial cells) where it resides in a host-membrane-derived vacuole termed the inclusion (Abdelrahman and Belland, 2005; Elwell et al., 2016). The EB converts into the replicating reticulate body (RB), which divides and eventually differentiates back into the EB form. EBs exit the host cell through cell lysis or inclusion extrusion [not pictured, (Hybiske and Stephens, 2007)] ~36–72 h postinfection. **(B)** *C*. *burnetii* infects macrophages where it resides within an acidified phagolysosome-like vacuole known as the *Coxiella*-containing vacuole (CCV) (Minnick and Raghavan, 2012). Both forms of *C*. *burnetii*, the small-cell and large-cell variants (SCV/LCV), are infectious with the SCV being more environmentally stable and having a lower metabolic rate than the LCV. *C*. *burnetii* exits the cell through cell lysis, although the timing of natural release is uncertain. For experimental usage, cells are artificially lysed once the bacteria reach the stationary phase after ~5–6 days. *C*. *burnetii* may also be grown axenically in acidified citrate cysteine medium [ACCM, (Omsland et al., 2009; Vallejo Esquerra et al., 2017)]. **(C)** Both *Anaplasma* spp. and *Ehrlichia* spp. have an infectious dense-core cell form (DC) and a replicative reticulate cell form (RC) (Salje, 2021). Cell types infected vary by species and include neutrophils, monocytes/macrophages, erythrocytes, or endothelial cells. Following internalization in a membrane-bound vacuole, the DC form converts into the RC form which then replicate as a bacterial microcolony known as a morulae. RCs eventually convert back to the DC form and are released from the cell ~3 days postinfection by either lysis (pictured), exocytosis, or filopodia in an actin-dependent mechanism. The exit mechanisms can vary by species and the stage of infection. **(D)** *Rickettsia* spp. replicate within the cytoplasm and most commonly infect endothelial cells, although some species will also infect macrophages. Members of the typhus group (TG) exit the cell via cell lysis (~4–5 days postinfection), whereas the spotted fever group (SPG) members can enter into adjacent cells via actin-based motility (depicted) or be released following lysis of the host cell (~48 h postinfection). **(E)** *Orientia* spp. infect a wide variety of cells including endothelial cells, dendritic cells, monocytes, and macrophages (Salje, 2021). Bacteria replicate within the cytoplasm in close proximity to the nucleus and are released from the cell in a budding process ~4 days postinfection. Note that for all of the bacteria above, the cell types listed reflect natural infection reservoirs and that multiple permissive cell types may be used in the lab for bacterial growth and propagation. In addition to the listed differences in time for bacterial entry, replication, and release, the infectious burdens per host cell vary across species from as little as ~30–50 for the SPG *Rickettsia* to a few hundred for *Chlamydia* spp. Images created with BioRender.com.

As obligate intracellular pathogens, these organisms place a significant burden on researchers when it comes to culturing options. Namely, with the exception of *C. burnetii* (Omsland et al., 2009), all require monoxenic growth in tissue culture. In general, they are all slow-growing pathogens requiring days to weeks for production of sufficient progeny to perform experiments and some exhibit developmentally distinct forms specialized for infection or replication. In addition, their intracellular location varies from the cytoplasm to neutral (pH) or acidified vacuoles. Collectively, these traits have a significant, complicating impact on genetic approaches. Considerations include which developmental form to use for mutagenesis, how to introduce nucleic acid, when to apply drug selection and which agent to use, and how to isolate clonal populations of mutants. In addition, the reduced genomes of these organisms (Andersson et al., 1998; Stephens et al., 1998; Seshadri et al., 2003; Brayton et al., 2005; Dunning Hotopp et al., 2006; Nakayama et al., 2008) make it difficult at times to discern whether a gene-inactivation approach has failed due to methodological shortcomings or gene essentiality. The host cell types infected, bacterial intracellular location and egress routes, and developmental stages targeted for mutagenesis are summarized in **Figure 1**. Development of axenic culturing conditions for other obligate intracellular pathogens, as achieved for *C. burnetii* (Omsland et al., 2009), would represent a significant technological advance for researchers. It is not a coincidence that the greatest number of genetic tools and largest mutant collection exists for *C. burnetii*.

### Mutant generation

Construction of mutants most commonly occurs through the introduction of DNA that will either be maintained in trans as a plasmid or integrated into the chromosome concomitant with or without the loss of native DNA. Plasmids have been useful for providing genes in trans and have been used to generate fluorescent protein marked strains, to express epitope-tagged proteins enabling tracking of protein localization, to manipulate gene expression via CRISPR interference (CRISPRi), and for complementation of chromosomal mutants, as examples. Plasmid-recombinant strains are typically easier to obtain than recombinant chromosomal mutants assuming that both options are available for the species. Use of plasmids as gene delivery vehicles does require an understanding of plasmid-maintenance mechanisms for the respective organism, and while broad-host-range plasmids do exist, the majority of plasmid success stories in the obligate intracellular bacteria have relied on the use of shuttle vectors constructed by merger of a native or closely related species plasmid with an *E. coli*-based vector. Important considerations for plasmid-expression approaches include the plasmid copy number, which can impact the strength of gene expression via gene dosage, and the impact that plasmid replication and accessory plasmid genes will have on the fitness of the bacterium. This is especially true for plasmids that are known to contribute to virulence. For example, the chlamydial native plasmid is found in most *Chlamydia* spp. and is known to affect virulence via plasmid-encoded genes and plasmid-based regulation of chromosomally encoded genes in a species-dependent manner (O’Connell et al., 2007; Carlson et al., 2008; Frazer et al., 2012).

Methods to construct bacterial chromosomal mutants include both random (e.g., transposon mutagenesis or chemical mutagenesis) and site-specific (e.g., allelic exchange, group II intron, lambda-red-mediated recombination, and CRISPR) approaches. These methods have been primarily used to disrupt gene function through insertional disruption or gene deletion, but they can also be leveraged to allow for gene expression at a single copy to alleviate gene-dosage concerns that arise when using plasmids. All chromosomal-gene disruption approaches come with the risk of introducing polar effects whereby the gene mutation alters the expression of neighboring genes, typically 3′ to the mutation. Stability of the chromosomal gene mutation over repeated passages or in animal/insect infection models where selection may not be easily applied can also be a concern depending on the approach used and is a problem that also extends to plasmids.

Due to their reduced genomes, attempts to make mutations in essential genes will lead to failure to obtain mutants or only transient existence of the mutant (Cheng et al., 2013; O’Neill et al., 2021). Published methods for obligate intracellular bacteria to address the contribution of essential genes to growth and virulence include gene silencing via antisense RNA, via peptide nucleic acids, or through the use of CRISPR interference (CRISPRi) [(McClure et al., 2017), expanded upon below]. Alternatively, conditional–lethal approaches should be feasible through introduction of an expression-regulated gene copy, typically plasmid encoded, followed by disruption of the chromosomal copy of the gene.

#### Introduction of DNA/RNA

There are multiple approaches for introducing nucleic acids into bacteria including physical methods (electroporation/gene gun), chemical methods (CaCl_2_ transformation, PAMAM dendrimers, or liposomes), and biological approaches (transduction [phage], conjugation, and natural transformation) (Parsons, 2013). The approach used will vary based on the species targeted and the desired outcome. Variables to consider include the developmental form of the bacterium being used, the efficiency of the process, and biological constraints (e.g., absence of known phage or phage receptors will limit transduction approaches). Electroporation, chemical transformation, dendrimer-enabled transformation, and natural transformation have all been documented for the obligate intracellular bacteria with electroporation being the most broadly applicable, and although quantifying DNA uptake is not readily feasible outside of *C. burnetii*, the overall efficiency of DNA introduction appears to be universally low and typically requires the use of high concentrations of DNA (microgram) compared with other bacteria (nanogram).

#### Chemical and UV irradiation mutagenesis methods

In lieu of nucleic acid-based approaches, mutants can also be obtained using chemical- or irradiation-based approaches. Both approaches are random in nature, will affect both plasmids and the chromosome, typically generate multiple mutations per chromosome, and often require laborious back-end work to sort mutants through screens or whole-genome sequencing of individual clones as selectable markers are not used. However, these approaches should be applicable to all obligate intracellular bacteria including those that genetic tools are not yet available for such as *O. tsutsugamushi*. Efforts in *Chlamydia* have been particularly illuminating regarding the power of chemical mutagenesis using EMS (ethyl methanesulfonate [GC to AT transitions]), ENU (N-ethyl-N-nitrosourea [AT to TA transversions and AT to GC transitions]), or NTG (N-methyl-N′-nitro-N-nitrosoguanidine [G:C to A:T transitions]) for creating temperature-sensitive mutants and for both forward and reverse genetic approaches (Kari et al., 2011; Nguyen and Valdivia, 2012; Rajaram et al., 2015; Longbottom et al., 2018). While not reported for the obligate intracellular bacteria, mutation via UV irradiation has also been used to mutagenize bacteria through induction of translesion DNA synthesis (Murli and Walker, 1993). In addition to these approaches being broadly applicable since transformation and selection barriers are removed, mutations within open-reading frames (preferably non-sense or non-conserved missense mutations) are often non-polar.

### Mutant selection and screening approaches

Selection of mutants for obligate intracellular bacteria is currently based on the use of antibiotic resistance directed by the selection markers available for the species. In other bacteria, auxotrophic requirements have been used for selection in addition to the classical use of resistance markers and here we report new auxotrophic approaches for *C. burnetii*. Unlike many other bacterial pathogens, antibiotic resistance is not a widespread problem among obligate intracellular pathogens owing in part to a paucity of naturally occurring resistance genes likely due to limited direct interactions with other bacteria. The general absence of resistance markers has made the choice of markers an empirical process, taking into account a number of species-specific considerations including the number of biological membranes that the drug must penetrate, the activity profile of the drug in acidified vacuoles if present, the rate of development of spontaneous resistance, bacterial drug sensitivity, and avoidance of clinically used drugs (Kamaruzzaman et al., 2017). In addition, bacteria with developmental forms (**Figure 1**) require careful selection of promoters along with the timing of drug delivery to ensure that the resistance marker is expressed both prior to and throughout selection. Screening approaches have not been widely employed in the obligate intracellular bacteria but in other bacteria generally utilize colorimetric (ex. blue-white screening on LB agar X-Gal [5-bromo-4-chloro-3-indolyl-β-D-galactopyranoside] plates) or fluorometric (ex GFP-expression) methods, or other gross-phenotypic changes such as altered colony morphology (Link et al., 2007).

### Isolation and expansion of clones

Genetic studies typically require starting with a clonal population. Traditionally, clones would be obtained by colony growth on agar plates. However, colony isolation is only currently applicable to *C. burnetii*. Other approaches that have been useful for obtaining clones under monoxenic conditions include plaquing, limiting dilution, isolation via micromanipulation, and fluorescence-activated cell sorting (FACS). The utility of these approaches is organism dependent and also remains reliant on the genetic methodology used when creating the mutant. For example, FACS cloning would be most effective with fluorescent-protein-expressing bacteria whereas limiting dilution requires that infectious bacterial progeny can be easily separated from one another.

In the following sections, we will expand on these topics as they relate to each pathogen.

## *Chlamydia* spp.

### DNA uptake, antibiotic selection, and clone isolation

Evidence that DNA uptake should be possible in *Chlamydia* stemmed from the early detection of *C. trachomatis* clinical isolates bearing *ompA* (encodes the major outer membrane protein, MOMP) that appeared to be recombinants between different serovars (Lampe et al., 1993; Hayes et al., 1994). Whole-genome sequencing of a large number of clinical isolates has further supported that *C. trachomatis* can exchange DNA during natural infections (Jeffrey et al., 2010; Joseph et al., 2012; Putman et al., 2013). Experimental evidence for lateral gene transfer was first provided by Demars and confirmed by multiple later studies (Demars et al., 2007; DeMars and Weinfurter, 2008; Suchland et al., 2009; Suchland et al., 2019). These studies used *Chlamydia* strains carrying different resistance alleles to coinfect cells. Drug selection was then applied to select for isolates carrying combinations of the resistance alleles presumably mediated by DNA exchange and recombination among the coinfecting strains. While the mechanism of DNA exchange was not defined at the time, transformation was proposed as the most likely mechanism. The ability of *Chlamydia* to carry out natural transformation has received support from a *comEC* transposon mutant that displays reduced natural or chemical transformation efficiency (discussed below) (LaBrie et al., 2019). Generation of recombinant strains via coinfection remains a useful tool for the field, particularly for allele segregation in chemically mutagenized isolates (Nguyen and Valdivia, 2012; Brothwell et al., 2016).

Non-natural transformation of *Chlamydia* spp. with foreign DNA was first performed using electroporation in two independent studies using *C*. *trachomatis* or *C. psittaci* (Tam et al., 1994; Binet and Maurelli, 2009). The electroporation (two times at 1.6 kV, 600 ohms, 25 µF) of *C. trachomatis* yielded a strain that transiently carried an *E. coli*/*C. trachomatis* shuttle vector (Tam et al., 1994). The *C. psittaci* study led to the construction of a chromosomal mutant carrying a synthetic 16S rRNA allele (Binet and Maurelli, 2009). Use of electroporation has not been further reported for *Chlamydia*. Polyamidoamine (PAMAM) dendrimers, hyperbranched polymers of uniform size that form a type of nanocarrier that due to their cationic charges can bind DNA molecules, have been used for transformation of *C*. *trachomatis* and *C*. *pneumoniae* (Gerard et al., 2013; Kannan et al., 2013). Although both electroporation and PAMAM dendrimers have been subsequently utilized with other obligate intracellular bacteria with electroporation being the primary approach, CaCl_2_-based chemical transformation as first reported by Wang et al. has become the sole method used for transforming *Chlamydia* spp (Wang et al., 2011). Chemical transformation has been reported for *C*. *trachomatis*, *C*. *muridarum* (Liu et al., 2014; Song et al., 2014), *C*. *pneumoniae* (Shima et al., 2018), *C*. *felis* (Shima et al., 2018), *C. psittaci* (Shima et al., 2020), and *C*. *caviae* (suicide vector only for *C. caviae* (Filcek et al., 2019),). Both chemical transformation and electroporation are performed with the elementary body (EB) form of *Chlamydia* (**Figure 1**; Binet and Maurelli, 2009; Wang et al., 2011), whereas the PAMAM dendriplexes were used to deliver the DNA directly to the reticulate body (RB) form within infected cells (Kannan et al., 2013). Comparison of the published qualitative transformation efficiencies with PAMAM dendrimers versus electroporation/chemical transformation indicate that PAMAMs are much more efficient and include a report of successful PAMAM transformation of a shuttle vector in the absence of selection. However, use of PAMAMs has only been reported by one lab, and although the efficiency of chemical transformation based on qualitative analysis using either electroporation or chemical transformation appears to be low on a per EB basis, successful transformation at the population level has become fairly routine using the chemical method.

All current chemical transformation protocols stem from Wang et al., whereby the mixing of microgram levels of DNA with CaCl_2_ and EBs is followed by a 30-min incubation and subsequent infection of cells using centrifugation (Wang et al., 2011). Most labs use unmethylated DNA for transformation, as *Chlamydia* spp. genomes appear to lack DNA methylases (Stephens et al., 1998), and early transformation studies reported that DNA prepared from methylation-deficient *E. coli* (Dam^-^/Dcm^-^) was more efficient than methylated DNA (Binet and Maurelli, 2009) or that unmethylated DNA was required for transformation (Wang et al., 2011). Use of methylation-deficient *E. coli* strains should only be used for production of DNA for direct use in transformation and not for plasmid construction or routine propagation, as DNA mutations are more common in methylation-deficient strains. Choice of the host cell for selection and propagation of transformants differs among labs with the most common cell lines being HeLa, Vero, McCoy, L2, or HEp-2 cells with the HEp-2 cells primarily used for *C. pneumoniae*. As with the other obligate intracellular bacteria, excepting *C. burnetii*, axenic culturing conditions have not been developed for *C. trachomatis*, although there are two medium types that support some metabolic activity outside of the host cell (Omsland et al., 2012; Grieshaber et al., 2018).

Antibiotic selection is added at different time points postinfection depending on the antibiotic and species used, with timing typically occurring after at least 8 h and before 24 h to allow for EB to RB conversion but before widespread EB production, respectively. The former allows for phenotypic conversion through resistance marker expression, and the latter prevents contamination of harvested bacteria with non-transformed EBs that are non-susceptible to drug selection. Antibiotics used for selection of recombinant strains transformed with resistance markers include ampicillin (*bla*; *C. trachomatis*, *C*. *muridarum*, and *C. psittaci* (Wang et al., 2011; Song et al., 2014; Shima et al., 2020)), spectinomycin (*aadA*; *C*. *trachomatis* and *C*. *muridarum* (Lowden et al., 2015; Cortina et al., 2019)), chloramphenicol [*cat*; *C. trachomatis*, *C. muridarum*, *C. pneumoniae*, *C. felis*, and *C. caviae* (Xu et al., 2013; Filcek et al., 2019; Wang et al., 2019)], and blasticidin (*sh ble*, *C. trachomatis* (Ding et al., 2013)). Kasugamycin has also been used in combination with spectinomycin to select for a *C. psittaci* mutant, although this study used a synthetic 16S rRNA to confer resistance after allelic exchange with the chromosomal 16S rRNA gene rather than a resistance marker (Binet and Maurelli, 2009). Spectinomycin, ampicillin, and chloramphenicol are most commonly used. Expression of antibiotic resistance genes is typically controlled by the promoter that is native to the cassette, although the *Neisseria meningitidis porA* promoter (Wang et al., 2011) and the chlamydial *incDEFG* promoter have also been used (Cortina et al., 2019).

In most instances, bacteria that are transformed with DNA encoding a fluorescent protein marker can be detected upon one to two passages using fluorescence microscopy. Fluorescent clones can then be isolated as free EBs using FACS (Vromman et al., 2014) or through direct inclusion picking using a micro-manipulator (Chiarelli et al., 2020a). More common techniques for obtaining clones are the plaque assay (Banks et al., 1970) or limiting dilution (Mueller and Fields, 2015). The plaque assay is more time consuming than limiting dilution (~1 to 2 weeks), and not all species or strains plaque. It is also worth noting that the genes required for plaquing are not defined, although it is likely a multifactorial process involving a combination of growth rate, EB release, and infectivity. Consequently, certain mutations may cause a recombinant strain to become plaque deficient. In this regard, alterations in plaquing phenotypes have been leveraged to build libraries of chemically mutagenized *C. trachomatis* L2 (Kokes et al., 2015). Analysis of clones to confirm presence of a chromosomal mutation typically involves PCR amplification and sequencing of the allele of interest or whole-genome sequencing, which is becoming increasingly common due to the small genome size of *Chlamydia* spp. and the quickly evolving next-generation sequencing approaches leading to rapid turnaround time, reduced cost, and straightforward genome assembly. For plasmid-bearing recombinant strains, the plasmid is typically re-isolated from the chlamydial transformant and either the recombinant locus or the whole plasmid is sequenced.

### Modification of genomic DNA

Modifications of the chlamydial chromosome have been performed using transposon mutagenesis, group II intron insertional mutagenesis, allelic exchange, and chemical mutagenesis. Details on the two mainly used chemical mutagenesis approaches can be found here (Kari et al., 2011; Nguyen and Valdivia, 2013), whereas the sections below will focus on methods that utilize exogenously delivered DNA. It is important to note that chemical mutagenesis has provided numerous insights into chlamydial biology and chemical approaches are universally applicable to both genetically intractable and non-axenically culturable bacteria.

#### Himar1-mediated transposition and group II intron-insertional mutagenesis

**Figure 2:**
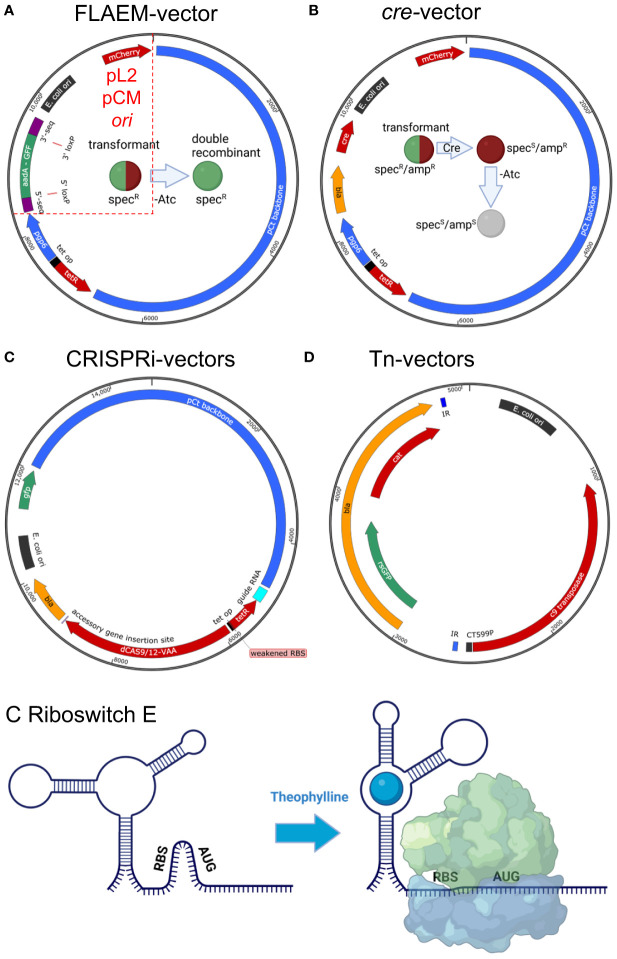
Updates to the chlamydial genetics toolbox. **(A)** Allelic exchange (FLAEM) utilizes the pSUmC-4.0 vector to obtain recombinant gene deletion mutants (Keb and Fields, 2020). 5′- and 3′- gene sequences from the targeted gene are cloned flanking the resistance gene (*aadA*) and reporter gene (*gfp*) which are expressed from their own promoters. Upon transformation, the strain exhibits red and green fluorescence and resistance to spectinomycin. Removal of aTc to halt *pgp6* expression creates a suicide plasmid scenario promoting homologous recombination under spectinomycin selection. A double-recombination event will result in a strain with green fluorescence and spectinomycin resistance. A single-recombination event is noted by a red and green fluorescent bacterium. Mini-shuttle vectors would only possess sequences defined by the red dashed lines and would include the native plasmid origin of replication (Fields et al., 2022). **(B)** The *cre*-bearing vector, pSU-CRE, can then be transformed into the strain and selected via ampicillin yielding a red and green fluorescent strain resistant to spectinomycin and ampicillin. Expression of Cre will mediate excision of the *loxP-*flanked *aadA*-GFP cassette generating a spectinomycin sensitive, red fluorescent bacterium. Removal of aTc can then be performed to cure the plasmid allowing for complementation of the mutant or construction of additional mutants. The cured strain will be sensitive to ampicillin and not fluorescent. **(C)** A base CRISPRi vector is shown with the guide RNA location shown in light blue (Ouellette et al., 2021). Guide expression is constitutive owing to a *dnaK* ^P^, and expression is isolated from downstream elements *via* an rrnB1 terminator sequence. Either d*cas9* or d*cas12* can be used for gene silencing, and expression of the dCas9/12 encoding an SsrA VAA degradation tag is controlled by the tet promoter. Accessory genes may be inserted 3′ of the d*cas* gene to make transcriptional fusions for complementation studies. **(D)** For transposon mutagenesis, a pUC-based suicide vector is used carrying the *himar1* c9 transposase and either *bla* [used for *C*. *trachomatis* (LaBrie et al., 2019)] or *gfp* and *cat* [for *C*. *muridarum* (Wang et al., 2019)] in the transposon region defined by the inverted repeats (IR, shown in blue). **(E)** A generic riboswitch is shown to represent the synthetic riboswitch E, which is responsive to theophylline (Grieshaber et al., 2022). In the absence of theophylline, the 5′ UTR folds to block the RBS. Binding of theophylline changes the folding of the 5′ UTR revealing the RBS and allowing for binding of the ribosome and translation. To create a “tighter” expression system, the riboswitch can be inserted downstream of the tet promoter (tet systems are shown in **A–C**) to allow for gene regulation via aTc induction and translational regulation via theophylline (not pictured). Vector maps were drawn with SnapGene, and sizes are approximate. Vectors are not drawn to scale. Images were drawn with BioRender.com.

Transposon mutagenesis using the Himar1 Mariner transposon has been universally applicable across the genetically tractable obligate intracellular bacteria to generate libraries of mutants carrying randomly inserted transposons. Success for *C. trachomatis* L2 was first reported in 2017 by Fischer et al. for a *cdu1*::Tn *bla* mutant (Fischer et al., 2017). This study determined that the Tn mutant lacking the *Chlamydia* deubiquinating enzyme (Cdu1) showed reduced infection in a mouse trans-cervical infection model, but the Tn method was not provided. Details on the Tn method were provided in 2019 by LaBrie et al. (2019), which described the use of a pUC19-derived suicide vector, plasmid *Chlamydia* Mariner (pCMA), carrying both the Himar1 C9 hyperactive transposase and the transposon possessing the *bla* gene cloned from the chlamydial shuttle vector pSW2 (Wang et al., 2011) (**Figure 2**). Transposase expression was driven by *ct599* ^P^ (serovar D nomenclature) including the *ct599* ribosomal binding site (RBS), which had been previously shown to have activity in *C. trachomatis* L2 while displaying limited activity in *E. coli*. 15 µg of plasmid was transformed into *C. trachomatis* L2, and mutants were selected with ampicillin. A total of 105 transposon mutants were obtained from 23 individual transformation experiments. The low number of mutants per transformation was associated with poor transformation efficiency rather than low transposase efficiency as parallel transformations with the replication competent vector pGFP::SW2 yielded similar numbers of wells with resistant bacteria. Transposon insertions were mapped with whole-genome sequencing, and the insertions occurred in the expected T/A position with no strains carrying double insertions. Key findings from the study indicated that most mutants showed minimal reduction in growth in cell culture, a few select mutants showed significant reduced infectivity in a mouse transcervical infection despite minimal growth differences in cell culture, and that inactivation of the putative ComEC homolog *ct339* resulted in a 100-fold reduction in lateral gene transfer efficiency during coinfection with two different serovars. The *ct399*::Tn *bla* strain could also not be transformed with pTLR2-GFP. The latter two findings support that *Chlamydia* are naturally competent and that competency is mediated in part through the activity of CT339. Improvement of DNA uptake by manipulating competence machinery could be a novel approach for increasing transformation efficiency, which appears to be a major bottleneck for chlamydial genetics.

Use of the Tn system was extended to *C*. *muridarum* by Wang et al. (2019). The pCMC5M transposon/transposase vector used a similar plasmid chassis as pCMA except that the *bla* marker was replaced by an *rsgfp*/*cat* cassette to enable selection with chloramphenicol and screening for green fluorescence. 10 µg of plasmid was used for transformations, and 33 chloramphenicol-resistant/GFP-expressing mutants were obtained. In contrast to the *C*. *trachomatis* Tn mutants that all had single insertions, four *C. muridarum* mutants had double Tn insertions. Some of the Tn mutants were complemented using a pNigg-mCherry (*bla*-marked) shuttle vector allowing for fulfillment of molecular Koch’s postulates. In addition, the Tn insertion was proven to be stable over at least 10 passages in cell culture without antibiotic selection.

While the Himar1 system works in at least two chlamydial species, the effort required to obtain mutants is prohibitive for constructing a saturated mutant library. In an attempt to circumvent the transformation efficiency barrier, Skilton et al. attempted to introduce the Himar1 system into *C. trachomatis* L2 on a chlamydial shuttle vector with the transposase under control of the anhydrotetracycline (aTc)-inducible *tet* promoter (Skilton et al., 2021). This approach would allow for isolation of a transformant that could then be expanded and subjected to Tn mutagenesis yielding a larger library, as all bacteria in the population carry the Tn vector. Attempts to deliver the functional system containing both the transposase and transposon on the same vector or on two separate vectors were unsuccessful. Data from the study suggest that leaky expression of the transposase is toxic for *C. trachomatis*, likely resulting in failure to obtain transformants. Long-term expression of the transposase would not have occurred in the prior studies that used a suicide vector. Coupling of accessory regulatory elements such as the theophylline riboswitch to the *tet* promoter, use of a suboptimal RBS sequence or alternate start codon for the transposase, and/or addition of the trans-translation SsrA-degradation tag to the transposase could reduce the “leakiness” of transposase expression in this system. In fact, addition of a riboswitch to a tet-inducible promoter allowing for transcriptional and translational control improved the utility of the Himar1 system (O’Neill et al., 2021). These regulatory mechanisms are further described below.

Insertional mutagenesis using a mobile group II intron was first reported for *C. trachomatis* in 2013 (Johnson and Fisher, 2013). This technique relies on the retargeting of the Ll.LtrB group II intron that contains an intron-targeting region and antibiotic resistance cassette for selection of the intron insertion. Targeting sequences for the intron are determined using an algorithm that can be built in-house based on (Perutka et al., 2004) or accessed through http://www.targetrons.com/ or http://www.clostron.com/. Intron expression has been driven by *incD* ^P^ (*C. trachomatis* (Weber et al., 2016c)) or *ctl0655* ^P^ [*C. trachomatis*, *C. muridarum*, and *C. caviae,* (Johnson and Fisher, 2013; Filcek et al., 2019; Cortina et al., 2022; Dolat et al., 2022)]. Detailed methodologies and construct information for group II mutagenesis have been reported (Key and Fisher, 2017; Weber and Faris, 2019). The GII intron approach has been the most widely adopted method for generating targeted-insertion chlamydial mutants and has been used in *C. trachomatis* (Johnson and Fisher, 2013; Weber et al., 2016c; Sixt et al., 2017; Almeida et al., 2018; Cosse et al., 2018; Panzetta et al., 2019; Ende and Derre, 2020), *C*. *muridarum* (Cortina et al., 2022; Dolat et al., 2022), and *C. caviae* (Filcek et al., 2019). For mutant construction, the intron is delivered on a suicide vector and insertion mutants are selected based on the drug marker encoded within the intron (*cat*, *bla*, and *aadA* have been used). Insertions have proven to be stable upon passage in cell culture and in mouse (Lowden et al., 2015; Cortina et al., 2022; Dolat et al., 2022) and chicken embryo infection models (Filcek et al., 2019) without selection. The choice of multiple selection markers allows for construction of multiple insertions in the same strain (Lowden et al., 2015) and enables complementation with chlamydial shuttle vectors (Weber et al., 2016c). Like all insertional approaches, polar effects must be considered and ideally introns should be targeted to the 5′ end of genes to maximize the chance of disrupting gene function. The group II intron method has also been used in *E. chaffeensis* and *R. rickettsii* (Cheng et al., 2013; Noriea et al., 2015).

#### Allelic exchange and Cre-loxP-mediated gene deletion

Allelic exchange identified via DNA sequencing of isolates obtained from natural coinfection or experimental coinfection supported that *Chlamydia* spp. were recombination proficient. Further support was provided by Binet et al. who performed an important proof-of-principle experiment in which a synthetic 16S rRNA allele encoding resistance to kasugamycin and spectinomycin was electroporated into *C. psittaci* (Binet and Maurelli, 2009). Dual-resistant mutants were obtained, and DNA sequencing revealed that the wild-type 16S rRNA had been replaced by the synthetic allele. In 2016, Mueller et al. provided the first generalized tool for constructing targeted gene deletions via recombination, which they termed Fluorescence-Reported Allelic Exchange Mutagenesis, or FRAEM (Mueller et al., 2016). For this approach, they used an *E. coli*–*C. trachomatis* shuttle vector, pSU6, modified to be a conditional suicide vector as the essential plasmid maintenance gene *pgp6* is regulated by the *tet* promoter. Homologous sequences (~3 kb) for the targeted gene are cloned 5′- and 3′- to *gfp bla* cassettes, and the vector also contains *mCherry*. Upon successful transformation in the presence of aTc to promote plasmid maintenance, the transformant would have red (mCherry) and green (GFP) fluorescence and be ampicillin resistant. Removal of aTc stops plasmid replication and requires plasmid integration into the genome for resistance to be maintained. Recombination at both the 5′- and 3′- homologous sequence regions results in loss of all plasmid elements including *mCherry*, while retaining the *gfp* and *bla* genes yielding an ampicillin-resistant, GFP-expressing mutant (Wolf et al., 2019). FRAEM is particularly useful in discriminating between transformation failure and failure to obtain a recombination/insertion mutant, as the transformation step is segregated from the mutagenesis step. FRAEM can also help assess gene essentiality through analysis of whether only a single recombination event (red and green bacteria) versus a double recombination event occurs (green only).

In addition to the usefulness of pSU6 for allelic exchange, it can also be used to cure *C. trachomatis* of its native plasmid and can be used to transiently express a gene of interest followed by plasmid removal when desired by omitting aTc from the culture (Mueller et al., 2016). The latter approach was used to develop a markerless deletion strategy known as FIoxed cassette allelic exchange mutagenesis, or FLAEM, to reduce the risk of polar effects and to allow for marker recycling (Keb et al., 2018; Keb and Fields, 2020). For the initial FLAEM approach, Keb et al. included *loxP* sites flanking the *gfp-bla* screening/selection markers used in the FRAEM system. In the updated approach, the pSUmC-4.0 base vector replaces the *bla* gene with *aadA* (Keb and Fields, 2020) (**Figure 2**). After a double-recombination event has occurred, the green fluorescent and spectinomycin-resistant strain is then transformed with a pSU variant carrying the *cre* recombinase (pSU-CRE) and *bla*/*mCherry* for selection and screening of transformants. In the presence of aTc, the vector is maintained allowing for expression of Cre recombinase and removal of the *loxP*-flanked chromosomal cassette. Removal of aTc then leads to vector curing and a non-resistant, non-fluorescent deletion mutation carrying only the *loxP* scar sequence. This allows for introduction of a vector for complementation or the construction of additional gene mutations within the same strain. The FRAEM/FLAEM approach has now been employed for multiple genes and in different labs (McKuen et al., 2017; Ghosh et al., 2020; Bui et al., 2021; Keb et al., 2021).

Collectively, the transposon mutants, chemical library non-sense mutants, and targeted gene inactivation mutants have indicated that a larger number of genes than were perhaps anticipated are non-essential at least for growth in cell culture. However, it seems likely that many genes dispensable for growth in cell culture will prove essential for growth in the animal or human host. With that in mind, it is important to be mindful of culturing conditions and how they impact both mutant selection and one’s assessment of mutant fitness.

### Shuttle vectors and regulated gene expression

The numerous shuttle vectors for *Chlamydia* spp. originate from the seminal study performed by Wang et al. (2011) and are widely used in the field. The two primary chlamydial vector backbones are p2TK2-SW2 (pSW2) (Wang et al., 2011) or pBOMB4 (pL2) (Bauler and Hackstadt, 2014). Replication-competent vectors have been used for a variety of purposes including complementation of mutants (Chen et al., 2014; Weber et al., 2016c; Panzetta et al., 2019; Luis et al., 2023), expression of epitope-tagged proteins for localization and secretion studies (Weber et al., 2015), as a platform for large-scale protein-partner identification via proximity labeling (Rucks et al., 2017), expression of reporter genes (Agaisse and Derre, 2013), and identification of effector proteins (Andersen et al., 2021; Yanatori et al., 2021). Shuttle vectors do show serovar and species tropisms (reports of serovar tropisms vary as highlighted by (Song et al., 2014; O’Neill et al., 2018)), with the species tropism for *C. trachomatis* and *C. muridarum* plasmids linked to CDS 2 (Wang et al., 2014). Consequently, shuttle vectors typically contain an *E. coli* replication competent backbone and accessory genes inserted into the native plasmid from the respective species targeted for transformation. Shuttle vectors were first developed for *C. trachomatis* [*bla*, *cat*, *sh ble*, and *aadA* (Wang et al., 2011; Ding et al., 2013; Xu et al., 2013; Mueller et al., 2016)] followed by *C. muridarum* (*bla*, *cat*, and *aadA* [Song et al., 2014; Wang et al., 2014; Cortina et al., 2019)] and more recently have been reported for *C. pneumoniae* [cat (Shima et al., 2018)], *C. felis* (*cat* (Shima et al., 2018)), and *C. psittaci* [*bla* (Shima et al., 2020)], which covers the majority of human pathogen-relevant or model-relevant *Chlamydia* spp. excepting *C. caviae*. The use of an *E. coli* backbone in combination with the native plasmid leads to large plasmid sizes (>10 kb), which can complicate downstream cloning and likely reduce transformation efficiency. As an alternative approach, Fields et al. devised a mini-replicon shuttle vector (base size of 5.45 kb) that contains only the native plasmid origin of replication and demonstrated that it can be maintained in the presence of the native plasmid under antibiotic selection in both *C*. *trachomatis* (serovars L2 and D) and *C*. *muridarum* (Fields et al., 2022). The copy number of the shuttle vectors does seem to differ from the native plasmids and varies between species and across plasmid backbones, although all plasmids are low-copy.

Expression of genes from the shuttle vectors has relied on the use of foreign promoters or chlamydial promoters. Native developmentally regulated promoters such as the early gene promoter *ihtA^P^* or the late gene promoters *omcA^P^* or *hctA^P^* have been used for developmental stage-specific expression of fluorescence reporter genes to track chlamydial development using live microscopy approaches (Cortina et al., 2019; Chiarelli et al., 2020b). Two inducible regulation systems have been published for *Chlamydia* and include the aTc-inducible *tet*-promoter system (Wickstrum et al., 2013) and the theophylline-inducible synthetic riboswitch E (Grieshaber et al., 2021; Grieshaber et al., 2022). The *tet*-promoter system has been shown to be functional in *C. trachomatis*, *C. muridarum*, and *C. psittaci* (Wickstrum et al., 2013; Cortina et al., 2019; Shima et al., 2020) and has been used for inducible gene expression in a mouse infection model with aTc-supplemented water (Cortina et al., 2022). Spacing of the TetR operator binding sites relative to the RBS has been found to impact the tightness of the system (Cortina et al., 2019). To provide an additional regulation approach, Grieshaber et al. developed a translational control system based on the synthetic riboswitch E whereby the riboswitch is used in place of the gene’s native 5′ UTR and RBS (Grieshaber et al., 2021; Grieshaber et al., 2022) (**Figure 2**). Addition of theophylline, which binds to the riboswitch, leads to opening of the mRNA, revealing the RBS and allowing for translation initiation. The study further showed that the riboswitch could be used in tandem with the *tet* promoter to allow for regulation of transcription and translation, increasing the tightness of the system (Grieshaber et al., 2022). Additional approaches to regulate the amount of proteins produced by shuttle-vector-encoded genes have included using a weakened promoter (Wickstrum et al., 2013) or a suboptimal RBS (Ouellette et al., 2021), or addition of an SsrA-degradation tag (Himeno et al., 2014) sequence to the gene, which results in degradation of the tagged protein by the ClpXP protease system (Ouellette et al., 2021).

#### Gene silencing

The ability to conditionally repress expression of genes is a critical tool for studying the function of essential genes. Such tools are even more relevant for the obligate intracellular bacteria, as many of the genes that remain in their reduced genomes are likely essential. The first report of gene silencing was for *C. trachomatis* and used an antisense approach using PAMAM dendrimers to deliver the antisense DNA primers to RBs in infected cells (Mishra et al., 2012). Follow-up studies using antisense methods have not been reported. More recently, Ouellette in two separate publications describes the development of CRISPRi for conditional gene silencing in *C. trachomatis* (Ouellette, 2018; Ouellette et al., 2021). The original report showed that *incA* could be knocked down using the catalytically “dead” Cas9 (dCas9) from *Staphylococcus aureus* under control of the *tet* promoter along with constitutive expression of a guide RNA (gRNA) targeted to the 5′ UTR of *incA* (Ouellette, 2018). To address problems with the first iteration of the CRISPRi system, namely, leaky expression of dCas9 and plasmid instability, Ouellette et al. switched from the pL2-LtetO vector backbone to the pBOMB vector backbone, weakened the dCas9 RBS sequence to reduce translation initiation efficiency, and added the SsrA-degradation tag (VAA) to dCas9 to reduce protein levels (Ouellette et al., 2021) (**Figure 2**). Importantly, Ouellette et al. also demonstrated not only knockdown of *incA* expression but that the knockdown could be complemented by making a transcriptional fusion between dCas9 and *incA*. Complementation is an important consideration to address concerns about off-target silencing by the gRNA and to assess for polar effects when silencing operon-encoded genes. In addition to supporting the efficacy of dCas9 for gene silencing, the study also found that the *Acidaminococcus* dCas12 could silence *incA*. gRNAs for the optimized CRISPRi were targeted to the 5′ UTR of *incA* with the dCas9 gRNA targeted to the template strand and the dCas12 gRNA targeted to the non-template strand. In theory, either strand should work for targets in the 5′ UTR whereas targets within the coding region should be on the non-template strand (Qi et al., 2013). As dCas9 and dCas12 have different protospacer adjacent motif (PAM) sequences (NNGRRT versus TTTV), having two options greatly increases the chances of finding a PAM sequence in the gene of interest. Indeed, Ouellette et al. report that almost 80,000 sites are present in *C. trachomatis* L2 when summing the dCas9 and dCas12 PAM sites (Ouellette et al., 2021). One other report on the use of CRISPRi in *C. trachomatis* L2 found toxicity associated with the expression of dCas9 proteins from *Streptococcus pyogenes* or *Staphylococcus aureus* when expressed in the absence of a guide RNA (Wurihan et al., 2019). Similar toxicities were not observed in the Ouellette studies, which used constitutive expression of a gRNA or the “empty” vector lacking the gRNA. Of note, the follow-up Ouellette study did employ tightly controlled dCas9/dCas12 protein levels using the SsrA-tagged constructs and a weakened RBS (Ouellette et al., 2021). Collectively, the reports do highlight that use of CRISPRi requires careful attention to both the levels of and the species source for the dCas9 protein. The CRISPRi approach has been used in multiple publications demonstrating its utility for use with both non-essential and essential genes (Wood et al., 2019; Wood et al., 2020; Brockett et al., 2021), and, in principle, CRISPRi should be feasible for the other obligate intracellular bacteria.

## Coxiella burnetii

### DNA uptake, antibiotic selection, and clone isolation

The first report of *C. burnetii* transformation was carried out using the virulent Nine Mile phase I clone 7 (NMI) strain (Suhan et al., 1996). Bacteria were isolated from infected BHK-21 cells and purified following mechanical lysis and centrifugation. Isolated bacteria were then washed in electroporation medium (10% glycerol or 10% glycerol/272 mM sucrose), and 0.5 µg of plasmid DNA was added. This mixture was then electroporated at either 12.5 or 16 kV for 6, 12, or 24 milliseconds. All subsequent *C. burnetii* transformations involved the use of electroporation to initiate DNA uptake. Bacteria used for transformation were isolated from either embryonated egg sacs (Lukacova M et al., 1999) or Vero cells (Beare et al., 2009) or grown in axenic media (Omsland et al., 2009; Omsland et al., 2011; Sandoz et al., 2016). Three different electroporation media have been used, 272 mM sucrose/10% glycerol (Beare et al., 2009), 270 mM sucrose (Chen et al., 2010), and 10% glycerol (Voth et al., 2011), whereas the amount of plasmid DNA used varied from 1 to 10 µg. In all studies to date, the following four electroporation conditions have been used: 1.6 kV, 500 Ω, and 25 µF (Beare et al., 2009); 2.0 kV, 500 Ω, and 25 µF (Beare et al., 2009); 1.8 kV, 500 Ω, and 25 µF (Omsland et al., 2011); and 2.5 kV, 400 Ω, and 25 µF (Chen et al., 2010). Three antibiotics have been approved for use in selecting transformants in *C. burnetii*, ampicillin (*bla*), kanamycin (*aphA*), and chloramphenicol (*cat*). Ampicillin and kanamycin have been used at 250–400 µg/ml, whereas chloramphenicol has been used at 3–5 µg/ml. Isolation of clonal *C. burnetii* transformants has been carried out using two different methods. The first method used micromanipulation to harvest single *Coxiella*-containing vacuoles (CCVs) from infected host cells (Beare et al., 2007). These isolated vacuoles were then expanded on new host cells and clonality determined by PCR. The second method that has been most widely utilized is the plating of transformants on semisolid axenic media (Omsland et al., 2011).

The potential for natural competence in *C. burnetii* has been discussed, as genes encoding components of a competence system are present in the genome (Seshadri et al., 2003). Due to the presence of frameshift mutations in three competence genes, *comA* (CBU0855), *comF* (CBU0464), and *comM* (CBU2022), it is unlikely that *C. burnetii* can naturally take up extracellular DNA.

### Modification of genomic DNA

The first transformation experiment performed in *C. burnetii* was described by Suhan et al. (1996). Using electroporation, the pSKO(+)1000 plasmid containing a previously discovered *C. burnetii* autonomous replicating sequence (ARS) (Suhan et al., 1994) and *bla* gene (for ampicillin resistance) was introduced into the bacterium (Suhan et al., 1996). This plasmid appeared to both autonomously replicate and, in some instances, integrate into the chromosomal copy of the ARS by homologous recombination. Four years later, the presence of the *bla* gene product β-lactamase in transformed *C. burnetii* was further confirmed using immunoblot analysis (Suhan and Thompson, 2000). These seminal findings provided hope for future genetic modification of *C. burnetii* in showing that ampicillin could be used as a selectable marker and that homologous recombination was possible. A subsequent study using a plasmid containing the *bla* gene, *C. burnetii* ARS, and GFP under the control of the *E. coli trp*/*lac* promoter was used to express GFP in *C. burnetii* (Lukacova M et al., 1999).

The development of an axenic medium for *C. burnetii* has resulted in a rapidly expanding genetic toolbox (Omsland et al., 2009; Omsland et al., 2011; Sandoz et al., 2016) both by making it easier to work with the bacteria and isolate clones and by increasing the number of genes amenable to manipulation since the repertoire of genes essential or highly important for growth on host-free medium is fewer than for growth in cell culture. One is also more likely to obtain high growth/virulence-attenuated strains on axenic solid media than in cell culture, as highly attenuated mutants growing as an individual colony do not have to compete with more robust mutants for resources or survive in a host cell. These parameters should yield a more diverse transposon mutant library comparing axenic to cell culture mutagenesis conditions.

#### Himar1-mediated transposition

The first described genetic mutants in *C. burnetii* were presented by Beare et al. (2009). This breakthrough was achieved using Himar1 transposition delivered using a two-plasmid system. The first plasmid (pCR2.1-P1169-Himar1C9) contained the *himar1* C9 allele, whose expression was driven by the CBU1169 small heat shock promoter, whereas the second plasmid (p1898-Tn) contained the Himar1 transposon consisting of the *mCherry* and chloramphenicol (*cat*) genes under the control of the CBU1169 promoter. Following electroporation, the bacteria were used to infect host cells where selection of transformants was achieved using chloramphenicol. This study identified 35 Himar1 insertions in the *C. burnetii* Nine Mile RSA439 phase II (NMII) genome; of these, 30 were intragenic (including two insertions in the native QpH1 plasmid) and five were intergenic. Transposon insertion in the end of the cell division gene *ftsZ* resulted in perturbation of its function, causing a filamentous form, and decreased the overall bacterial growth rate in host cells.

Two studies were published in 2011 using Himar1 transposition in *C. burnetii* grown in axenic media to create mutants to the Dot/Icm-type IVB secretion system (T4BSS) genes *icmL.1* (CBU1629) (Carey et al., 2011) and *icmD* (CBU1624) (Beare et al., 2011). These two studies both confirmed that the Dot/Icm T4BSS system is essential for growth of *C. burnetii* in host cells and were only feasible owing to axenic growth. The next four publications using Himar1 transposition in *C. burnetii* generated transposon libraries for screening of potential virulence factors (Weber et al., 2013; Martinez et al., 2014; Newton et al., 2014; Metters et al., 2023). These libraries consisted of >20 (Weber et al., 2013), 3840 (Newton et al., 2014), >3000 (Martinez et al., 2014), and 10,000 (Metters et al., 2023) individual transposon insertions. Three libraries were generated using a single Himar1 plasmid system (Weber et al., 2013; Newton et al., 2014; Metters et al., 2023), whereas the third library was generated using a two plasmid Himar1 system (Martinez et al., 2014). Weber et al. described the identification of 20 transposon insertions in T4BSS effector genes, 10 of which had defects in intracellular growth and CCV formation (Weber et al., 2013). Newton et al. identified transposon insertions that resulted in defective T4BSS function, regulation of T4BSS expression, and T4BSS effector protein production as well as non-T4BSS-related insertions that affected *C. burnetii* growth and displayed various CCV phenotypes (Newton et al., 2014). Further research was carried out on one T4BSS effector, *cig2* (CBU0021). This effector was shown to be important for interaction between the CCV and host autophagosomes. Martinez et al. isolated >3,000 transposon insertions, 1,082 of which were sequenced and examined using a high-content multi-phenotype microscopy screen (Martinez et al., 2014). Three different aspects of *C. burnetii* infection were examined: internalization of the bacteria in host cells, vacuole formation and bacterial replication, and protection of infected host cells from apoptosis. Further analysis was performed on the transposon insertion in *ompA* (CBU1260). This transposon mutant displayed a defect in *C. burnetii* internalization and replication in host cells. OmpA-coated latex beads were able to be taken up by non-phagocytic host cells, and *C. burnetii* uptake was prevented when host cells were incubated with purified OmpA or *C. burnetii* was pretreated with an anti-OmpA antibody. These data helped to identify OmpA as the first *C. burnetii* invasion. The most recent mutant library garnered >10,000 individual mutants and is the first study to look at identifying essential genes in *C. burnetii* (Metters et al., 2023). A total of 511 genes were predicted to be essential.

The Himar1 transposon insertions generated by the aforementioned laboratories have been used in at least 28 subsequent publications (Norville et al., 2014; Winchell et al., 2014; Martinez et al., 2015; Latomanski et al., 2016; Martinez et al., 2016; Weber et al., 2016b; Weber et al., 2016a; Fielden et al., 2017; van Schaik et al., 2017; Bitew et al., 2018; Clemente et al., 2018; Crabill et al., 2018; Bitew et al., 2019; Kuba et al., 2019; Mansilla Pareja et al., 2019; Wachter et al., 2019; Bitew et al., 2020; Brann et al., 2020; Burette et al., 2020; Kuba et al., 2020; Martinez et al., 2020; Newton et al., 2020; Padmanabhan et al., 2020; Pechstein et al., 2020; Hofmann et al., 2021; Siadous et al., 2021; Steiner et al., 2021; Case et al., 2022). Below, we will highlight some of the findings using these transposon mutants. Most of these studies have focused on defining the role of T4BSS apparatus and/or effector proteins in host cell infection. Specifically, *C. burnetii* effector proteins have been shown to interact with host innate immune signaling (Burette et al., 2020), autophagy pathways (Martinez et al., 2016; Crabill et al., 2018; Siadous et al., 2021), and host trafficking pathways (Latomanski et al., 2016) and/or were essential for CCV biosynthesis (Martinez et al., 2015; Weber et al., 2016b; Martinez et al., 2020). Furthermore, the use of different T4BSS apparatus mutants has been used in many studies to define where *C. burnetii*’s T4BSS effector cohort is important within the context of host cell infection and to help define new T4BSS effectors (Weber et al., 2016a; Clemente et al., 2018; Mansilla Pareja et al., 2019; Brann et al., 2020; Padmanabhan et al., 2020; Steiner et al., 2021).

*C. burnetii* has a reduced metabolic capacity, presumably due to its shift to an intracellular lifestyle and reduced genome size (Seshadri et al., 2003). The development of an axenic medium and random Himar1 mutagenesis has allowed the study of the *C. burnetii* metabolic genes. Nicotinamide adenine dinucleotide (NAD) is a coenzyme central to metabolism; mutation of *nad* (CBU0101) in *C. burnetii* resulted in an intracellular growth defect and decreased levels of NAD, NADH, and NADP. *C. burnetii* can transport and metabolize glucose and glutamate into intermediates of glycolysis, gluconeogenesis, and the TCA cycle (Kuba et al., 2019). *C. burnetii* encodes at least two potential hexose transporters (CBU0265 and CBU0347). Using Himar1 mutants in these two genes, CBU0265-Tn and CBU0347-Tn, Kuba et al. were able to confirm their role in transporting glucose (Kuba et al., 2019). Using Himar1 mutagenesis, other *C. burnetii* genes have been identified as having roles in central carbon metabolism, including CBU0823 that contains both malate dehydrogenase and malic enzyme activities and SdrA (CBU1276), which is a short-chain dehydrogenase that facilitates regeneration of NADP(H) (Bitew et al., 2020). Recently, Wachter et al. identified a small RNA, CbsR12, that when mutated by Himar1 transposition resulted in the downregulation of *carA* translation, which encodes an enzyme that catalyzes the first step of pyrimidine biosynthesis, and upregulation of *metK*, which encodes an s-adenosylmethionine synthetase that is a part of the methionine cycle (Wachter et al., 2019).

All current Himar1 libraries have been generated in the avirulent NMII strain of *C. burnetii*. The historical *in vivo* model for *C. burnetii* infection has been the guinea pig (Moos and Hackstadt, 1987). Because NMII is avirulent in the guinea pig model (Moos and Hackstadt, 1987), two new *in vivo* models were developed for testing virulence in NMII Himar1 mutants. The first model developed was created using the larvae of the greater wax moth *Galleria mellonella* (Norville et al., 2014). This model was tested using the NMI, NMII, and NMII T4BSS apparatus mutants (*dotA*::Tn and *dotB*::Tn). Within this model, NMI and NMII were equally virulent whereas *dotA*::Tn and *dotB*::Tn were not, indicating that T4BSS is essential for survival of NMII. An attenuated virulence in this model was observed for the previously described NMII *ompA*::Tn mutant (Martinez et al., 2014), indicating this model can allow intermediate virulence phenotypes to be studied. The second model developed used a severe combined immunodeficiency disease (SCID) mouse (Weber et al., 2016b; van Schaik et al., 2017). This model was tested using NMII and four Himar1 mutants (*dotA*::Tn, *cvpB*::Tn, *ompA*::Tn and *enhC*::Tn). In this model, NMII was virulent whereas the four Himar1 mutants displayed a range of attenuated virulence with *dotA*::Tn, *cvpB*::Tn, and *enhC*::Tn having no detectable splenomegaly whereas *ompA*::Tn had intermediate splenomegaly, consistent with the phenotype observed in the *G. mellonella* model (Martinez et al., 2014).

#### Allelic exchange and Cre-loxP-mediated gene deletion

Sixteen years following the first example of homologous recombination in *C. burnetii* (Suhan et al., 1996), Beare et al. were able to generate the first site-directed gene deletions (Beare et al., 2012). Two strategies were used to create mutants in the T4BSS apparatus genes *dotA* and *dotB*. The first strategy used a loop-in/loop-out allelic exchange method to replace the *dotA* and *dotB* genes with the chloramphenicol-resistance cassette, *cat*. A suicide vector containing *cat* surrounded with 5′- and 3′- flanking regions of the target genes was first integrated into the genome by homologous recombination. A second round of homologous recombination using sucrose counterselection resulted in deletion of the target gene. A detailed protocol for this procedure has been published (Beare and Heinzen, 2014). The second strategy used Cre-*lox*-driven recombination to delete the *dotA* gene. This method required the integration of two plasmids containing *loxP* sites and different antibiotics 5′- or 3′- of the *dotA* gene. Introduction of a third suicide plasmid, encoding the Cre recombinase, and using sucrose counterselection resulted in deletion of *dotA*. The loop-in/loop-out strategy has been used to create 24 other mutants in *C. burnetii* (Larson et al., 2013; Beare et al., 2014; Cunha et al., 2015; Larson et al., 2015; Colonne et al., 2016; Vallejo Esquerra et al., 2017; Stead et al., 2018; Larson et al., 2019; Moormeier et al., 2019; Pechstein et al., 2020; Schäfer et al., 2020; Friedrich et al., 2021; Sanchez and Omsland, 2021; Sandoz et al., 2021; Clemente et al., 2022). Many of these mutants have targeted components of the T4BSS, including 10 T4BSS effectors (*icaC*, *cvpA*, *cvpB*, *cvpC*, *cvpD*, *cvpE*, *cpeD*, *cpeE*, *ankF*, *ankG*) (Larson et al., 2013; Cunha et al., 2015; Larson et al., 2015; Colonne et al., 2016; Pechstein et al., 2020; Schäfer et al., 2020), the T4BSS chaperone IcmS (Larson et al., 2019), and PmrA, the regulator of T4BSS apparatus and effector expression (Beare et al., 2014).

Site-directed mutants (using the loop-in/loop-out approach) have also helped to identify five genes essential for LPS biosynthesis in *C. burnetii* (Beare et al., 2018), the first known virulence factor for this organism. This study included the first reported deletion mutants in the virulent NMI bacteria. Morphological differentiation plays a large part in *C. burnetii* development. Three publications using deletion mutants have started to delve into the differences between the large-cell and small-cell *C. burnetii* developmental forms. Mutation of an outer membrane phospholipase A prevents stationary phase modifications to membrane lipids (Stead et al., 2018), whereas deletion of the stationary phase sigma factor *rpoS* resulted in downregulation of stress response and peptidoglycan remodeling genes (Moormeier et al., 2019). Recently, Sandoz et al. discovered that in bacteria such as *C. burnetii* that lack the Braun’s lipoprotein, the bacteria instead use outer membrane proteins to tether the outer membrane to peptidoglycan in the periplasm (Sandoz et al., 2021). Utilizing deletion mutants, Sandoz et al. were able to show that this cell-cycle-dependent tethering to the outer membrane protein BpbA was partially dependent on L,D-transpeptidases. The DotA mutant generated by Beare et al. (2012) has subsequently been used to show the role of T4BSS in endosomal maturation (Samanta et al., 2019), inhibition of neutrophil apoptosis (Cherla et al., 2018), inhibition of NF-kB and RelA activation (Mahapatra et al., 2016), and inhibition of B-cell pyroptosis (Schoenlaub et al., 2016) and in recruiting actin patches on the CCV (Miller et al., 2018).

**Figure 3:**
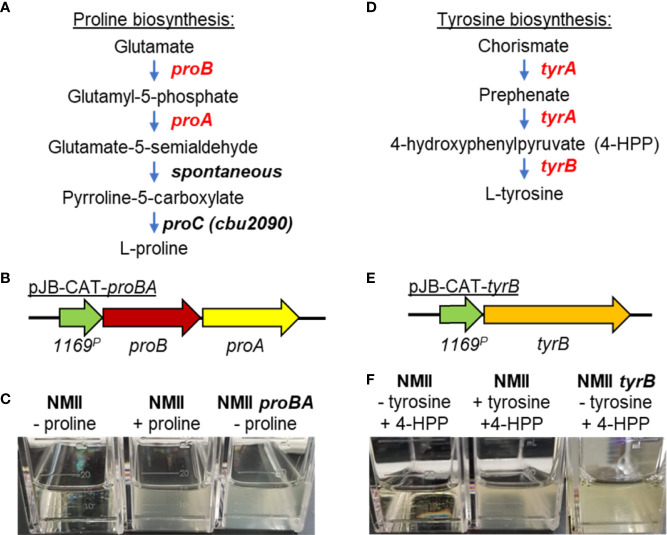
Complementation of *C*. *burnetii* proline and tyrosine auxotrophies for transformant selection. **(A)** Pathway of proline biosynthesis from glutamate. *C*. *burnetii* has lost the *proA* and *proB* genes that catalyze the first two steps of proline biosynthesis from glutamate while retaining *proC*, which catalyzes the final step in the proline biosynthesis pathway. **(B)** Schematic of the *proBA* operon cloned from *Legionella* and placed under the control of the CBU1169 promoter. The *1169^P^*-*proBA* operon was cloned into the pJB-CAT shuttle vector and the plasmid transformed into *C*. *burnetii*. **(C)** Complementation of the proline auxotrophy was tested using ACCM-D plus or minus proline. Expression of *proBA* in NMII (NMII *proBA*) complemented growth of the bacterium in the absence of proline, whereas the wild-type bacteria (NMII) could only grow in the presence of added proline. **(D)** Pathway of tyrosine biosynthesis from chorismate. *C*. *burnetii* has lost both genes (*tyrA* and *tyrB*) required for biosynthesis of tyrosine from chorismate. **(E)** Schematic of the *tyrB* gene cloned from *E*. *coli* and placed under the control of the CBU1169 promoter. This fragment was then cloned into pJB-CAT and the plasmid (pJB-CAT-*tyrB*) transformed into *C*. *burnetii*. **(F)** Complementation of the tyrosine auxotrophy was tested using ACCM-D with the addition of the TyrB substrate 4-hydroxyphenylpyruvic acid (4-HPP) and plus or minus tyrosine. Expression of *tyrB* in NMII (NMII *tyrB*) complemented growth of the bacterium in ACCM-D containing 4-HPP but minus tyrosine, whereas the wild-type bacteria (NMII) could only grow in the presence of added tyrosine and not in the presence of the TyrB substrate 4-HPP alone.

The Cre-*lox* method was recently used to create a 32.5-kb deletion containing 23 of the 26 T4BSS apparatus genes (Long et al., 2021). This mutant was shown to be avirulent in guinea pigs and was protective as a whole-cell vaccine against challenge with virulent *C. burnetii*. While there have been >20 published site-directed gene deletions, these two methods have a limited use within the *C. burnetii* field. Many of these studies relied on the selection of mutants using antibiotic selection (carbenicillin, kanamycin, or chloramphenicol) in low-pH axenic media. Carbenicillin and kanamycin are sensitive to low pH, requiring very high concentrations (>250–400 µg/ml) for transformant selection. The development of a defined axenic medium has permitted the creation of nutritional selection based on *C. burnetii*’s natural auxotrophies to 11 amino acids. Complementation of the final steps in the arginine and lysine biosynthetic pathways using *argGH* and *lysCA* genes from *Legionella pneumophilia* allowed growth of *C. burnetii* transformants in ACCM-D in the absence of arginine and lysine, respectively. Here, we describe the development of two nutritional selection systems based on complementation of proline and tyrosine auxotrophies (**Figure 3**) (Wachter et al., 2022).

### Shuttle vectors, site-specific transposition, and regulated gene expression

Two different shuttle vectors have been described, pKM230 (Chen et al., 2010) and pJB-CAT (Voth et al., 2011). Both shuttle vectors are based on the *Legionella* plasmids pJB908 and pJB2581, respectively. The basis for the autonomous replication of these vectors in *C. burnetii* is directed by the RSF1010 origin. This origin of replication results in a copy number of approximately 3–6. These shuttle vectors have been used for a variety of different reasons including complementation of mutant strains (Bitew et al., 2018; Kuba et al., 2019), complementation of amino acid arginine and lysine auxotrophies and central metabolic deficiencies (Sandoz et al., 2016; Beare et al., 2018; Sanchez et al., 2021), reporter constructs for identifying both T4BSS-dependent (Chen et al., 2010; Voth et al., 2011) and T4BSS-independent (Stead et al., 2013) effector secretion (BlaM, CyaA, 3x FLAG fusions), and epitope tagging proteins of interest (Fielden et al., 2017; Justis et al., 2017) (3xFLAG, 2xHA) and for creating fluorescent strains (Chen et al., 2010; Martinez et al., 2014) (mCherry, GFP).

The native plasmid found in most *C. burnetii* is predicted to have one to three copies, for example the QpH1 plasmid in NMII, whereas some strains exist whereby the endogenous plasmid has integrated into the genome (Samuel, 1986; Savinelli and Mallavia, 1990). Recent studies have described the development of two new shuttle vectors, pQGk (Luo et al., 2021) and pB-TyrB-QpH1ori (Wachter et al., 2022). These two shuttle vectors both contain an *E. coli* origin of replication (pUC or pMB1, respectively), the origin of replication and replication genes from the endogenous *C. burnetii* plasmid QpH1 (CBUA0036, CBUA0037, CBUA0038, CBUA0039, and CBUA0039a), and selection in *C. burnetii* using either kanamycin or complementation of the tyrosine auxotrophy in tyrosine minus ACCM-D. The pB-TyrB-QpH1ori plasmid has a separate *cat* gene for chloramphenicol selection in *E. coli*. Introduction of these plasmids into *C. burnetii* with selection resulted in curing of the native QpH1 plasmid owing to the presence of the QpH1 origin in the shuttle vector. This QpH1-less NMII strain was further shown to have a growth defect in host cells.

**Figure 4:**
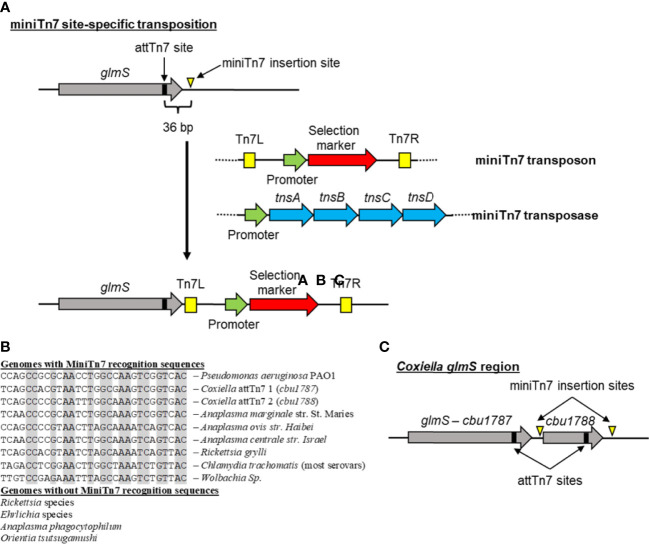
MinTn7 transposition in bacteria. **(A)** Schematic of site-specific miniTn7 transposition into the region downstream of the glutamine-fructose-6-phosphate gene (*glmS*). The *glmS* gene has a 30-bp attTn7 site in the end of it that is recognized by the miniTn7 transposase (consisting of the TnsA, TnsB, TnsC, and TnsD proteins). Subsequent miniTn7 insertion occurs 36 bp downstream of the attTn7 site. This transposition system utilizes two plasmids, one containing the miniTn7 transposon with a selectable marker and the second containing the miniTn7 transposase genes. **(B)** Alignment of predicted miniTn7 attTn7 sites in different obligate intracellular bacteria compared with *Pseudomonas aeruginosa*. No predicted attTn7 sites were identified in *Rickettsia*, *Ehrlichia*, or *Orientia* species and even though other *Anaplasma* species have an attTn7 site, these were not found in *A. phagocytophilum*. Gray boxes indicate conserved sequences. **(C)** Schematic of the *C*. *burnetii glmS* region. Due to a duplication event between the end of *cbu01787* and *cbu01788*, *C*. *burnetii* has two different attTn7 sites allowing for integration into either site.

Beare et al. (2011) described the use of a gDNA modification system using the site-directed miniTn7 transposon. The miniTn7 transposon uses a two plasmid-based system, one plasmid containing the miniTn7 transposon and the other containing the miniTn7 transposase (encoded by the *tnsABCD* genes). Initial reports identified the integration site as being downstream of CBU1787, with the miniTn7 recognition site (TCAGCCACGTAATCTGGCGAAGTCGGTGAC) located in the 3′ end of CBU1787. Subsequent analysis has identified a second miniTn7 recognition sequence (TCAGCCCCGCAATTTGGCAAAGTCGGTGAC) in the 3′ end of the CBU1788 gene resulting in the miniTn7 integration downstream of CBU1788 (**Figure 4**). Comparison of the CBU1787 and CBU1788 genes indicated that a duplication event of the last 63 bp of CBU1787 had occurred resulting in two potential miniTn7 recognition sites. This system of site-specific DNA integration is the main method used to complement both site-directed and Himar1 transposon mutants (Beare et al., 2009; Beare et al., 2012). The miniTn7 transposon has also been used to create a single-copy luciferase reporter system for *C. burnetii* that identified PmrA-dependent and -independent promoters (Beare et al., 2014).

**Figure 5:**
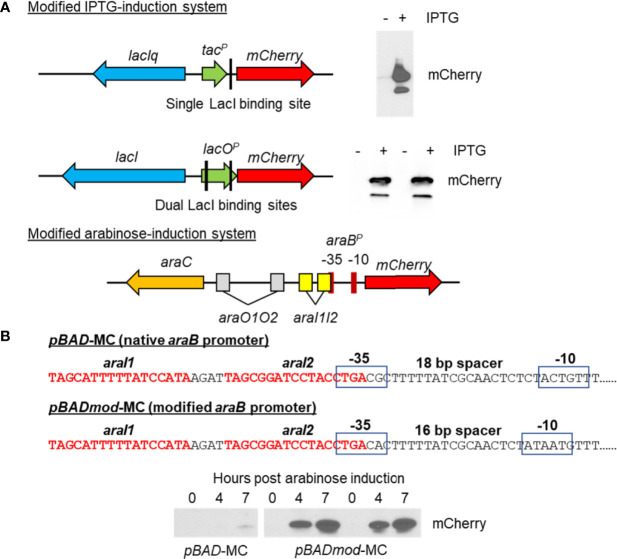
Creation of new IPTG and arabinose-inducible expression systems in *C*. *burnetii*. **(A)** Schematics of two different IPTG-induction systems allowing inducible expression of mCherry and their respective immunoblots detecting mCherry production. The upper schematic shows the original IPTG-inducible system contained on the pJB-CAT and pKM230 *C*. *burnetii* shuttle vectors, which consists of a truncated *lacI* gene (missing the tetramerization domain), a *tac* promoter, and a single LacI binding site upstream of *mCherry*. Production of mCherry was seen in the minus IPTG induction by immunoblot (upper right) indicating leaky expression of *mCherry*. The lower schematic shows the new IPTG-inducible system consisting of a full-length *lacI* gene and *lacO* promoter containing dual LacI binding sites upstream of *mCherry*. No background mCherry production was seen in the uninduced control, whereas good production of mCherry was evident in the IPTG-induced sample. **(B)** Schematic of the arabinose-induction system allowing inducible expression of *mCherry*. The *mCherry* gene was cloned downstream of the *araB* promoter. The sequence of the native *araB* promoter is shown. This sequence was modified so that the -35 and -10 regions had a smaller 16-bp spacer region and their sequences were closer to the consensus sigma 70 promoter sequence (TTGACA and TATAAT, respectively). The native and modified arabinose induction systems were cloned into the pJB-CAT shuttle vector and then transformed into *C*. *burnetii*. Arabinose-based gene expression was examined by immunoblot of 0-, 4-, or 7-h post-arabinose induction of 5-day *C*. *burnetii* cultures. Production of mCherry from the native *araB* promoter was very low at 7 h, whereas in the modified *araB* system, high levels of protein were detected at both 4 and 7 h post induction. No background mCherry production was seen in the uninduced control (0 h).

**Figure 6:**
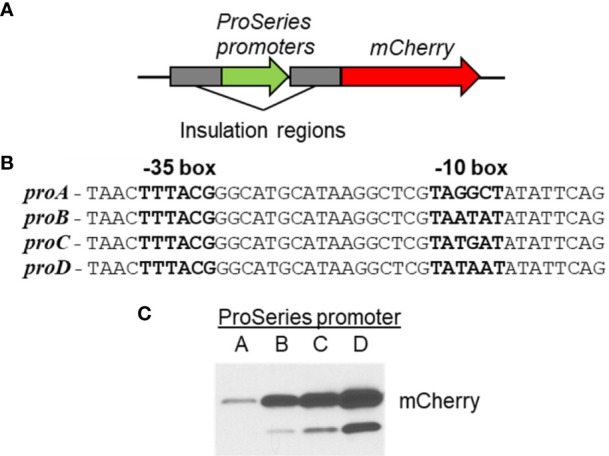
Creation of synthetic promoters for predictable gene expression in *C*. *burnetii*. **(A)** Schematic of the variable strength ProSeries synthetic promoters fused upstream of the *mCherry* gene. The synthetic promoters are flanked by insulation regions that help prevent unwanted gene transcription. **(B)** Sequence of the four different ProSeries promoters (*proA*, *proB*, *proC*, and *proD*) is shown. The only difference between each promoter lies in the -10 box. **(C)** The four different promoter-*mCherry* fusions were cloned into pJB-CAT, and the resulting vectors were transformed into *C*. *burnetii*. The level of promoter activity was examined by looking at mCherry production by immunoblot. An increasing level of mCherry production was seen across the four promoter constructs.

*C. burnetii* currently has two different methods for inducible gene expression (Chen et al., 2010; Beare et al., 2011), the *lac* operator/repressor and anhydrotetracycline systems. Both systems use a small-molecule (IPTG or anhydrotetracycline) to induce gene expression via the *tac* or *tetA* promoters, respectively. Here, we present a modified *lac* inducible system (**Figure 5**) that uses the *lac* promoter in place of the *tac* promoter and uses a full-length LacI containing the tetramerization domain, which the LacI produced by pKM230 and pJB-CAT shuttle vectors lacks. We also present a third system based on arabinose-inducible gene expression from the *araB* promoter (**Figure 5**). Only two promoters have been used for general constitutive gene expression in *C. burnetii*, *cbu1169* ^P^, and *cbu1910* ^P^. However, many complementation experiments have used gene-specific promoters to recapitulate normal expression. A synthetic promoter system has been developed for *C. burnetii* that allows for predictable and tunable constitutive gene expression using the ProSeries set of insulated promoters (Davis et al., 2011; Cooper et al., 2017) (**Figure 6**). Genetic manipulation of *C. burnetii*, aided by the use of axenic media, continues to progress toward the level seen for free-living bacteria.

## Rickettsiales

### Anaplasmataceae: *Anaplasma* and *Ehrlichia*

#### *Anaplasma phagocytophilum* and *marginale*: DNA uptake, antibiotic selection, and clone isolation

Transformation of A*. phagocytophilum* and *A. marginale* was described in 2006 and 2010, respectively (Felsheim et al., 2006; Felsheim et al., 2010). Isolated bacteria were used to infect either HL-60 or ISE6 cells to greater than 90%, respectively. The cells were then disrupted via needle aspiration and filtered and the bacteria washed in either 270 mM sucrose or 250 mM sucrose, respectively. The method of DNA uptake used for these two species was electroporation using 1 µg of each plasmid DNA mixed with bacteria in a 0.1-cm-gap cuvette. *A. phagocytophilum* and *A. marginale* used slightly different electroporation conditions with *A. phagocytophilum* using 1.2 kV, 400 Ω, and 25 µF, whereas *A. marginale* used 2.5 kV, 400 Ω, and 25 µF. Most research articles published since have described the use of electroporation to initiate DNA uptake. The most recent *A. phagocytophilum* and *A. marginale* transformations were achieved using slightly modified electroporation conditions, 1.7 kV, 400 Ω, and 25 µF (O’Conor et al., 2021) and 2.0 kV, 400 Ω, and 25 µF (Crosby et al., 2014), respectively. In both cases, the bacteria were washed in 300 mM sucrose. Selection of transformants has predominantly been via the use of spectinomycin and streptomycin resistance conferred by the *aadA* gene, and more recently gentamicin resistance has also been used (Hove et al., 2022). Interestingly, in a step toward non-antibiotic selection in *A. phagocytophilum*, Oliva Chavez et al. (2019) tested the use of the herbicide resistance *bar* gene, which provides resistance to phosphinothricin (PPT), for selection of transformed bacteria. Isolation of clonal mutants from Himar1 transposition experiments is achieved using limiting dilution. The development of an axenic media is underway with preliminary data showing both DNA and protein biosynthesis of bacteria isolated in host-cell free phagosomes (Zhang et al., 2021). This study provides a basis for further media modifications with the hope of creating an environment allowing bacterial replication.

A second method of DNA uptake by *A. phagocytophilum* was reported by Oki et al. (2015). This method used PAMAM dendrimers which can deliver the DNA directly to bacteria within infected cells (Mishra et al., 2012). Using this technique, *A. phagocytophilum* was transformed with dendrimers complexed with a single-plasmid DNA (pCis GFPuv-SS Himar A7) containing the Himar1 transposase and transposon (Oki et al., 2015). Transformants were obtained but failed to survive multiple passages, suggesting that the location of the transposon insertions was detrimental to *A. phagocytophilum* growth.

### Modification of genomic DNA

Genetic modification of the *Anaplasma* genus has been primarily carried out using the Himar1-based transposon system (Oki et al., 2015; Oliva Chavez et al., 2019; Eskeland et al., 2021; O’Conor et al., 2021). The initial *A. phagocytophilum* study utilized a dual-plasmid technique whereby the Himar1 transposase was expressed using the *A. marginale tr* promoter on the plasmid pET28AMTR-A7-HIMAR whereas the transposon, containing *tr*-promoter-driven expression of GFPuv and spectinomycin/streptomycin resistance, was present on the pHIMAR1-UV-SS plasmid (Felsheim et al., 2006). Once inside the bacteria, the active Himar1 A7 transposon binds and cuts the Himar1 transposon from the plasmid and inserts it randomly into the gDNA of the target genome at TA dinucleotide sequences. *E. coli* rescue cloning was used to identify the location of the transposon insertions and identified eight different Himar1 transformants. A subsequent *A. marginale* study by Felsheim et al. (2010) also used the pET28AMTR-A7-HIMAR plasmid for Himar1-A7 transposase production but modified the type of GFP gene used in the transposon plasmid pHIMAR1-UV-SS from GFPuv to TurboGFP (pHimarAm-trTurboGFP-SS). Himar1 transposition was not detected; instead, homologous recombination of the pHimarAm-trTurboGFP-SS plasmid was observed with the genome copy of the Tr promoter (Felsheim et al., 2010), indicating that homologous recombination in *A. marginale* is viable. Successful Himar1 transposition in *A. marginale* was not demonstrated until 4 years later when Crosby et al. (2014) utilized a single Himar1 plasmid that contained both the *himar1*-A7 transposase gene and Himar1 transposon (pHimarcisA7mCherry-SS). All previous transformation attempts using a two-plasmid Himar1 system were unsuccessful, hence a single Himar1 plasmid system was tested (Crosby et al., 2014), with the hypothesis that a bacterial cell has a higher probability of taking up a single plasmid than two plasmids. Transposon insertion site junctions were identified using Roche/454 and Illumina-based sequencing and confirmed by PCR. A single transposon mutant was identified in the *omp10* gene. This mutant was later shown to have reduced infectivity in cattle (Crosby et al., 2015), indicating that the generation of Himar1 transposon mutants is possible and can identify genes important for virulence. To date, there have been six studies using Himar1 transposition in *Anaplasma* to create random transformants (Felsheim et al., 2006; Crosby et al., 2014; Oki et al., 2015; Oliva Chavez et al., 2019; Eskeland et al., 2021; O’Conor et al., 2021) with the largest Himar1 transposon library reported (1,195 insertions) recently created by O’Conor et al. (2021). From these pools of Himar1 transformants, a further six research articles have been published (Noh et al., 2011; Hammac et al., 2013; Crosby et al., 2015; Oliva Chavez et al., 2015; Crosby et al., 2020) and have helped identify potential virulence genes, including an O-methyltransferase required for tick cell infection (Oliva Chavez et al., 2015), a putative effector protein (Oliva Chavez et al., 2019), a VirB6 paralog with attenuated growth in human and tick cell lines (Crosby et al., 2020), and a T4SS effector that is essential for tick infection (Park et al., 2023).

Recently, Oliva Chavez et al. (2019) analyzed transpositions of the Himar1 transposon expressing the *bar* gene, which confers resistance to the herbicide PPT, using PPT selection. In the absence of a second antibiotic-based selection marker, resistance to PPT via the *bar* gene will allow a second round of transformation in previously generated Himar1 mutant strains. Use of the *bar* marker should also allow complementation of mutants and enable the construction of double mutants.

Targeted mutagenesis in *Anaplasma marginale* has recently been achieved (Hove et al., 2022). In this study, the vector AM581-KO-*tuf*-mCherry-Gent, containing the tuf promoter-mCherry-gentamicin cassette flanked by 1.1 kb of 5′ and 3′ DNA that flank the AM581 gene, was used to a create linear PCR product that was used to delete the AM581 gene (encoding the phage head-to-tail connector protein) by allelic exchange. Selection of transformed bacteria was established using gentamicin selection driven by the *E. chaffeensis tuf* promoter and confirmed with subsequent visual analysis of mCherry production. Homologous recombination was achieved via a double recombination event. In a bovine infection model, this mutant “exhibited no clinical disease” and had an *in vivo* growth defect when compared with wild-type bacteria. The mutant was also tested as a modified live vaccine, displaying protection against challenge with virulent *A. marginale*, whereas use of wild-type *A. marginale* in an inactivated whole-cell vaccine did not provide protection. These data suggest that protection against bovine anaplasmosis may require not only the B-cell response but other immune mechanisms.

### Shuttle vectors and regulated gene expression

To date, there are no publications for the development of a shuttle vector for *Anaplasma* and native plasmids have not been identified in the *Anaplasma* spp. Control of all ectopic gene expression in the *Anaplasma* spp. has been achieved using the *Anaplasma marginale tr* promoter. The *tr* promoter is one of only a few *Anaplasma* promoters that have been analyzed and was found during the study of the *msp2* expression (Barbet et al., 2005). The *tr* promoter was chosen due to its activity in both mammalian and tick cells. To date, no inducible expression system has been developed for *Anaplasma*.

### *Ehrlichia chaffeensis, muris*, spp. HF: DNA uptake, antibiotic selection, and clone isolation

The first documented report of transformation of the *Ehrlichia* genus was in 2005 (Long et al., 2005). This study used electroporation to transiently transform *E. muris* with 1 µg of plasmid containing the chloramphenicol resistance gene under the control of a promoter from *E. chaffeensis*. Bacteria were isolated from infected DH82 canine monocyte cells and resuspended in 10% glycerol. Electroporation using a 0.1-cm-gap cuvette was carried out using the following conditions: 2.5 kV, 200 Ω, and 25 µF. All subsequent *Ehrlichia* transformations have been carried out using electroporation as the desired method for DNA uptake. The next reported study showing *Ehrlichia* transformation was not until 8 years later and was carried out using *E. chaffeensis* (Cheng et al., 2013). In this study, bacteria were isolated from infected DH82 cells (for targeted mutagenesis) or ISE6 cells (for random mutagenesis) using different procedures, whereas both final bacterial transformation mixes were resuspended in 300 mM sucrose as opposed to 10% glycerol. Bacteria were mixed with 1–6 µg of either linear PCR fragments or plasmid DNA in a 0.1-cm-gap cuvette and electroporated using the following conditions: 2.0 kV, 400 Ω, and 25 µF. Fourteen years following the first transient transformation of *E. muris*, Lynn et al. (2019) reported the use of electroporation to create *E. muris* genetic transformants. *E. muris* was isolated from infected cells, resuspended in sucrose, and mixed with 1 µg of each plasmid then electroporated using the same conditions as previously described for *A. phagocytophilum* (Felsheim et al., 2006). The *Ehrlichia* spp. HF strain was recently transformed using electroporation (Bekebrede et al., 2020). Bacteria were isolated from DH82 cells, resuspended in 300 mM sucrose, and mixed with 6–8 µg of plasmid DNA in a 0.2-cm-gap cuvette. Electroporation was carried out using the following conditions: 2.5 kV, 400 Ω, and 25 µF. Initial transformant selection was achieved using chloramphenicol, which proved a poor method of selection. Subsequent studies have switched to selection of transformants using spectinomycin and streptomycin via the *aadA* marker. More recently, selection of transformants using gentamicin has been reported (Wang et al., 2017). Isolation of clonal mutants from Himar1 transposition studies has been achieved using limiting dilution of infected cells. Initial studies have begun toward the development of an axenic medium for *Ehrlichia*. The first study found DNA and protein synthesis in purified reticulate cells (RCs), but not from dense-core cells (DCs), incubated in axenic media (Eedunuri et al., 2018). In a subsequent study, phagosomes containing *Ehrlichia* were used to examine DNA and protein biosynthesis (Zhang et al., 2021). These studies provide an excellent start toward the development of an axenic medium that supports growth of *Ehrlichia*.

### Modification of genomic DNA

The first reported case of genetic mutation of the *Ehrlichia* genus was described by Cheng et al. (2013). This study utilized three different methods to create genetic mutants in *E. chaffeensis*. The first method used linear PCR fragments, containing a chloramphenicol or gentamicin cassette (where *cat* and gentamicin expression was driven by the *E. chaffeensis rpsl* promoter) flanked by regions of the targeted insertion site, to test allelic exchange. Following electroporation, Southern blot analysis of PCR products confirmed that these linear fragments integrated into the *E. chaffeensis* genome at the target site by homologous recombination. These transformants unfortunately only survived up to 8 days and then were lost, suggesting the target sites may be essential for survival. The second method utilized a mobile group II intron-based mutagenesis method. To drive the expression of the group II intron, the *E. chaffeensis tuf* promoter (Ech_0407) was chosen whereas the expression of *cat* was driven using the *E. chaffeensis rpsl* promoter. Six different genomic locations, both intergenic and intragenic, were chosen. Group II intron insertion was confirmed in three locations, but as with the PCR-based homologous recombination, transformants lasted only up to 8 days in culture. The final technique described in the report used the Himar1 transposon system to create random *E. chaffeensis* transformants. A single-plasmid construct containing the *A. marginale tr* promoter drives *himar1 A7* expression and the transposon consisting of a fluorescent reporter (GFPuv or mCherry) and the streptomycin/spectinomycin antibiotic resistance gene (*aadA*). Following electroporation, transformants that were stable in culture for several months were analyzed and Himar1 transposition was confirmed by PCR, identifying nine transposon insertions. This study indicated that the use of the *aadA* resistance gene was better for selecting transformants than chloramphenicol or gentamicin, an important finding for future genetic studies in *Ehrlichia*. Subsequent studies have used Himar1 transformants generated in this study to show attenuated virulence in deer (reservoir host) and canines (incidental host) and polar effects on gene expression due to Himar1 insertion (Cheng et al., 2015). Furthermore, attenuated Himar1 mutants were able to induce protection against wild-type *E. chaffeensis* challenge in both reservoir and incidental host (Nair et al., 2015) and from tick-transmitted wild-type challenge in the canine host (Mcgill et al., 2016).

*E. chaffeensis* encodes a type IVA secretion system that allows transport of bacterial effectors into infected host cells (Alvarez-Martinez and Christie, 2009; Rikihisa, 2015). The first *E. chaffeensis* T4ASS effector (*etf-1*) was identified (Lin et al., 2011) and shown to be secreted (Liu et al., 2012). This effector has two functions, blocking host cell apoptosis (Liu et al., 2012) and inducing Rab5-regulated autophagy (Lin et al., 2016). A more recent study described the use of peptide nucleic acids (PNAs) to knock down expression of *etf-1* (Sharma et al., 2017). PNAs are synthetic mimics of DNA whereby the deoxyribose phosphate backbone is replaced with a pseudo-peptide polymer to which nucleobases are then linked. PNAs can form high-affinity and specific bonds with complementary DNAs or RNAs (Pellestor and Paulasova, 2004). The *etf-1* targeted PNAs electroporated into *E. chaffeensis* were able to decrease *etf-1* expression and show that *etf-1* was required for *E. chaffeensis* growth (Sharma et al., 2017). Furthermore, PNA-based *etf-1* knockdown resulted in reduced *E. chaffeensis*-mediated host cell autophagy. This study provided a methodology for targeting potentially essential genes in *E. chaffeensis* that cannot be obtained with traditional gene inactivation approaches.

As a follow-up to the first report of allelic exchange by the Ganta lab (Cheng et al., 2013), this lab also developed a modified allelic exchange method to create stable deletions of two individual genes, Ech_0230 and Ech_0379) (Wang et al., 2017). This vector contained ~1 kb of 5′ and 3′ flanking DNA to the genes of interest, and within these flanking regions was an antibiotic cassette containing the *tuf* promoter driving expression of the *aadA* gene. These vectors were then used to generate linear PCR products that were then transformed into *E. chaffeensis*. Selection of transformants resulting from double crossover events was then carried out by treating infected ISE6 tick cells with spectinomycin. Complementation of Ech_0379 was achieved using the same linear PCR allelic exchange methodology except that the linear fragment contained the complete Ech_0379 gene. Selection of complemented bacteria was achieved using a cassette containing the *A. marginale* Tr promoter driving expression of the gentamicin resistance gene. This study achieved two important milestones for allelic exchange in *E. chaffeensis*, first, the generation of stable targeted mutants, and second, complementation of targeted mutations to test molecular Koch’s postulates to define a gene function. The first genetic modification of *E. muris* was reported by Lynn et al. (2019) and used two separate single-plasmid Himar1 transposase/transposon constructs (pCis mCherry-SS HimarA7 and pCis mKate-SS HimarA7). Himar1 transposition generated eight different insertions, both intergenic and intragenic. No defect was observed in either tick or monkey endothelial cells for any of the Himar1 transformants. The *Ehrlichia* HF strain, unlike *E. chaffeensis*, is highly virulent for laboratory mice (Fujita and Watanabe, 1994). Due to its utility in mouse models of infection, Bekebrede et al. (2020) used Himar1 transposition to create 158 individual transformants, with 55 of these insertions located within coding regions. Two mutants, H34B and H59, had partially reduced virulence in mice. The most recent study using Himar1 transposition created 55 individual insertions in *E. chaffeensis* (Wang et al., 2020), with 31 of these being intragenic whereas the remaining 24 were intergenic. Testing of these transformants in a canine model found that 23 intragenic mutations were essential for persistence, whereas surprisingly 15 intergenic insertions also appeared essential for persistence. Furthermore, five Himar1 insertions that did not affect persistence in the canine model were unable to survive in the tick vector. Together, this study allowed the mapping of genes/genomic regions that are essential to both the host and/or tick reservoirs. Three further research articles have been published that continue to study previously generated Himar1 transformants: ECH_0379, insertion downstream of ECH_0490 and ECH_0660 (Kondethimmanahalli and Ganta, 2018; Kondethimmanahalli et al., 2019), and ECH_0660 and ECH_0665 (Torres-Escobar et al., 2021).

### Shuttle vectors and regulated gene expression

To date, there are no publications for the development of a shuttle vector for *Ehrlichia*. Whole-genome sequencing has also not detected the presence of a native plasmid in the *Ehrlichia* genome. Four different promoters have been used for ectopic expression in *Ehrlichia*. The first promoter used was from *Ehrlichia*, and it drove expression of the *cat* gene. However, the promoter was not identified so the nature of the promoter is unknown (Long et al., 2005). The remaining three promoters used (*rpsl* and *tuf* from *E. chaffeensis* and *tr* from *A. marginale*) were all described in a single study (Cheng et al., 2013). Each promoter was used in a different method for mutagenesis of *E. chaffeensis*. The *rpsl* promoter was used to drive the expression of *cat* from plasmid constructs used to make mutants by homologous recombination. Targeted mutations were obtained using a modified group II-intron-based system, whereby the expressions of the modified group II intron RNA, *cat* gene, and intron-encoded protein were driven by the *tuf* promoter. The final promoter that has been used has been the tr promoter from *Anaplasma*. This promoter was chosen because *A. phagocytophilum* is closely related to *E. chaffeensis* and was predicted to be functional in *Ehrlichia*. The *tr* promoter was used to drive expression of components of a two-plasmid Himar1 transposition system including the Himar1 transposase, mCherry or GFP, and *aadA* gene to confer resistance to spectinomycin and streptomycin. As with the *Anaplasma* spp., no inducible promoter system has been developed.

### *Rickettsiaceae: Orientia* and *Rickettsia*

#### *Orientia tsutsugamushi:* steps toward genetics

*O. tsutsugamushi* [formerly *Rickettsia tsutsugamushi,* (Tamura et al., 1995)], the causative agent of scrub typhus, remains on the list of genetically intractable organisms. *O. tsutsugamushi* can be grown in a variety of cell types in the laboratory with Vero cells (green monkey kidney epithelial cells) and L929 cells (mouse fibroblast) being the most commonly used, and detailed methods for culturing, quantifying, and storing the bacterium have been described (Giengkam et al., 2015). In addition to established cultivation methods, possible approaches for obtaining clones have been devised and include the plaque assay (strain-dependent (Hanson, 1987),) and the use of fluorescent dyes (Atwal et al., 2016) or Click-it chemistry to fluorescently label live bacteria (Atwal et al., 2020). The fluorescent labeling approaches could in theory be married to FACS to enable isolation of single bacteria as would be performed for a fluorescent-protein-expressing bacterium. Of note, as some strains plaque, it is possible that the chemical mutagenesis approaches used for *Chlamydia*, and recently in a forward genetics approach for the intractable and unculturable *Wolbachia* (Duarte et al., 2021), could be leveraged to obtain banks of growth-attenuated *Orientia* isolates, as illustrated for *C. trachomatis* (Kokes et al., 2015).

In a direct attempt to pave the way for genetic tools in *Orientia*, Hunt and Carlyon performed a promoter assessment study to identify a promoter that could be used to drive the expression of antibiotic resistance genes or reporter genes (Hunt and Carlyon, 2021). They sought promoters that were highly conserved across clinical and lab strains, would be expressed in a variety of cell types infected by *O. tsutsugamushi* (**Figure 1**), and would provide constitutive expression during infection with a robust transcription profile. Three promoters were assessed, *ompA*, *tsa22*, and *tsa56*, with the *tsa* genes possessing two putative promoters designated as up and down. All promoters chosen had sequence similarities with *E. coli* -10 and -35 sequences and included putative ribosomal binding sites. Expression of GFP in *E. coli* as a surrogate model was assessed with each promoter, and *tsa22^P^*-up yielded the greatest expression. The surrogate model approach has been used to identify promoters that proved useful for foreign gene expression in other members of the Rickettsiales (Policastro and Hackstadt, 1994; Barbet et al., 2005; Peddireddi et al., 2009). While all three promoters were expressed across different cell infection models, nucleotide sequence variation across strains was noted for *ompA^P^*, and the *tsa56^P^*-fl promoter (fl—full-length promoter with up and down sequences) had no activity in *E. coli*, leaving the prime candidates as *tsa22^P^*-down/fl and *tsa56^P^*-down/up. Of relevance to promoter selection, Atwal et al. describes the existence of a developmental cycle for *O. tsutsugamushi* with intracellular (IB) and extracellular (EB) bacterial forms (Atwal et al., 2022). If a developmental cycle does occur in *Orientia*, it could impact promoter choices as well as drug selection timing and the choice of bacterial form for DNA introduction.

For resistance marker selection, drugs currently used to treat scrub typhus include doxycycline (preferred), tetracycline, azithromycin, chloramphenicol, or rifampicin and thus markers providing resistance to these clinically used compounds should be avoided (Singh and Panda, 2021). Naturally occurring resistance markers for *Orientia* have been identified (Kelly et al., 2017), but they are generic in nature (multidrug efflux pumps) or not useful such as β-lactamase-encoding genes as *Orientia* are resistant to β-lactams due to the presence of a minimalistic cell wall (Atwal et al., 2017). Further susceptibility testing of antibiotics [or herbicides as shown for *Anaplasma* (Oliva Chavez et al., 2019)] could be useful in identifying a viable selection system for *Orientia* in combination with the promoters identified by Hunt and Carlyon (2021). It also seems likely, given the high AT content of *Orientia* [70% AT, (Batty et al., 2018)], that foreign genes will need to be codon optimized to enable efficient translation.

Plasmids have not been identified in *Orientia* (El Karkouri et al., 2016; Batty et al., 2018), although plasmids are found in the related *Rickettsia* and appear to have originated from *Rickettsia*/*Orientia* chromosomes (El Karkouri et al., 2016), indicating that the currently used rickettsial shuttle vectors could have applicability in *Orientia*. Moreover, while plasmids appear to be absent from the genus, mobile genetic elements are rampant providing evidence of lateral gene transfer within *Orientia* and supporting that mobile elements can be integrated into the chromosome (Batty et al., 2018; Fleshman et al., 2018). Though many of those mobile genes are degraded and functionality is unclear (Gillespie et al., 2012), some of them are expressed and appear to be regulated via antisense RNA, a regulatory process that appears to be widespread in *Orientia* (Mika-Gospodorz et al., 2020). The Himar1 transposon, which has proven to be successful in other obligate intracellular bacteria, would appear to be a likely candidate for generating mutants in *Orientia* if a selection system can be identified.

### *Rickettsia* spp. DNA uptake, antibiotic selection, and clone isolation

The vector-borne, pathogenic *Rickettsia* spp. are subdivided into three groups, the typhus group (TG), the spotted fever group (SFG), and the transitional group. A fourth grouping, the ancestral group, is non-pathogenic. The genetic tool kit for modifying *Rickettsia* spp. is fairly well established with transposon mutagenesis approaches and shuttle vectors available for most of the species along with documented usage of targeted gene inactivation methods [recently reviewed by (McGinn and Lamason, 2021)]. Nonetheless, obtaining mutants remains a laborious process owing to poor transformation efficiency and the isolation of clones is challenging due to slow growth and the absence of axenic culturing conditions. The status of some rickettsial species as risk group 3 agents requiring work in BSL-3 facilities or select agents (*R. prowazekii*) has also slowed genetic advances for logistical reasons rather than biological ones.

*R. prowazekii* and *R. typhi*, members of the typhus group (TG), were two of the earliest success stories for genetics in obligate intracellular bacteria with the demonstration of allelic exchange using a rifampicin resistant *rpoB* R546K allele in 1998 (Rachek et al., 1998) followed by construction of a GFP-expressing *R. typhi* strain in 1999 using an *rpoB^WT^-gfp_uv_* gene fusion (Troyer et al., 1999). Both of these studies utilized electroporation to transform bacteria freshly isolated from infected cells (L929 or Vero cells, respectively) and either rifampicin resistance or GFP expression to identify mutants. A similar approach to Troyer et al. (1999) was subsequently used to generate a GFP-expressing *R. conorii* mutant (Renesto et al., 2002). Electroporation remains the sole approach used for genetic manipulation of *Rickettsia* spp. and has been used to introduce DNA, DNA/transposase complexes, or peptide-nucleic acids. Four different electroporation conditions have been used. The first set of conditions were 1.7 kV, 129 Ω, 50 µF (Rachek et al., 1998) and 2.5 kV, 200 Ω, 25 µF (Troyer et al., 1999). The electroporation conditions used by Troyer et al. (1999) have subsequently been used in other studies (Baldridge et al., 2005; Clark et al., 2011b), whereas more recently two different conditions have been reported, 1.8 kV, 200 Ω, 25 µF (Burkhardt et al., 2011; Oliver et al., 2014) and 1.7 kV, 150 Ω, 50 µF (Riley et al., 2015; Riley et al., 2016), that use a lower field strength. Bacteria are typically prepared for transformation via sucrose washing after isolation from infected cells followed by mixing with DNA (microgram quantities) and electroporation. A unified electroporation protocol has been published (Riley et al., 2015), and although subtle variations continue to be used, usage of microgram amounts of DNA and high bacterial titers (>10^8^) are common across research groups to compensate for the low efficiency of transformation. Multiple host cell types have been reported for recovering *Rickettsia* mutants with Vero, L929, and ISE6 cells being the most predominantly used cells. As for growth of all obligate intracellular bacteria, cell type considerations focus on ensuring that bacterial growth will be supported.

While the first genetic success for *Rickettsia* (Rachek et al., 1998) utilized the rifampicin-resistant *rpoB* allele isolated from a rifampicin-resistant mutant *R. prowazekii* strain E derived using chemical mutagenesis (Balayeva et al., 1985), subsequent selection approaches have utilized more classical “gain-of-function” resistance markers. The most frequently used marker is *arr-2*, which confers resistance to rifampicin. Other published markers include *ereB* [erythromycin; selection requires a ratio of <20 bacteria per cell (Rachek et al., 2000)], *cat* (chloramphenicol), and *aadA* (spectinomycin). Promoters used to drive the production of resistance markers, reporter genes, or foreign genes include the rickettsial promoters *gltA^P^*, *rpsl^P^*, *ompA^P^*, *ompB^P^*, and *flg^P^* from *Borrelia* (Welch et al., 2012) and *tr1^P^* from *A. marginale* (Arroyave et al., 2021). Differences between promoter strengths have been reported as highlighted by *ompA^P^* outperforming *ompB^P^* for driving reporter and resistance cassettes (Baldridge et al., 2010b).

Isolation of clonal populations is primarily performed using one of three approaches. FACS is the fastest method and can be used with either fluorescent reporter gene-expressing strains (Driskell et al., 2016) or using fluorescent antibody labeling (Riley et al., 2015), although the latter can negatively impact infection of cells after clone isolation. Limiting dilution has also been used, although multiple isolation and passages are recommended to ensure clonality, which can lead to long wait times for mutant isolation (~45 days) depending on growth rate. Plaquing, as performed with some *Chlamydia* spp., can be used with members of the SFG which spread cell to cell via actin-based motility. Moreover, while axenic conditions have not yet been established for *Rickettsia* spp., genome-based computational analysis of Rickettsial metabolic pathways and transport abilities has identified 51 metabolites that are likely acquired from the host (Driscoll et al., 2017) and would be required as supplements for an axenic culture system.

### Modification of genomic DNA

#### Transposon mutagenesis

Transposon mutagenesis was first reported for *Rickettsia prowazekii* in 2004 (Qin et al., 2004) and has since been reported for SFG members *R. conorii* (Kim et al., 2019), *R. monacensis* (Baldridge et al., 2005), *R. montanensis* (Baldridge et al., 2010b), *R. rickettsii* (Clark et al., 2011b), and *R. parkeri* (Welch et al., 2012). While the initial transposon used was the Epicentre EZ::TN Tn5 transposome system which is delivered as a transposase/DNA complex (Qin et al., 2004), the majority of subsequent Tn mutants have been constructed using the Mariner Himar1 transposon (Liu et al., 2007). The Himar1 system can be delivered as either a single (transposase plus transposon) or a double-plasmid system that separates the transposase and transposon genes. While banks of mutants have been obtained, the largest collection thus far contains 106 mutants leaving it far short of the number required for saturation-based analyses such as Tn-seq (Lamason et al., 2018). The “high-content” yielding approach utilized multiple transformations of *R. parkeri* with the Himar1 Tn and a direct electroporation-to-plaque protocol followed by passaging of isolates obtained from abnormal-looking plaques. Numerous novel mutants were obtained from this study including genes not previously described as impacting infection and a T4SS component mutant, which could shed light on the role of the T4SS in rickettsial pathogenesis. The Himar1 Tn has also been used for chromosomal insertion of genes encoding epitope-tagged proteins (Welch et al., 2012) and reporter genes [for example, (Baldridge et al., 2005)], and for single-copy complementation of mutants (Clark et al., 2011a). Recent updates to the Himar system include the use of a pLOXHIMAR vector derived from the pCis mCherry-SS Himar1 A7 vector and includes *loxP* sites flanking the resistance marker to enable excision of the marker by Cre recombinase. Marker removal was not performed (Arroyave et al., 2021), so it is not clear if Cre-*lox* recombination is functional in *Rickettsia*. The vector can be used with *Rickettsia*, *Ehrlichia*, and *Anaplasma* with spectinomycin selection. The utility of Tn mutagenesis continues to expand with the use of a Tn5-based minitransposon termed *kkaebi* to obtain 53 *R. conorii* Tn mutants (Kim et al., 2019). Using an O-antigen synthesis mutant and a plasmid complemented strain, the study demonstrated that the production of Weil-Felix antibodies during infection requires the O-antigen. From a genetics standpoint, at least for *R. conorii*, chloramphenicol selection was found to be superior over rifampicin selection owing to the development of spontaneous resistance to rifampicin (1 × 10^-7^ PFU), which was not observed with chloramphenicol (<1 × 10^-8^ PFU). Use of *cat* as the Tn marker also enabled introduction of an *arr-2*-marked *E. coli*–*R. conorii* shuttle vector based on pRAM18dRGA previously shown to replicate in *R. conorii* (Riley et al., 2016).

#### Targeted genetic approaches—allelic exchange, GII introns, and peptide nucleic acids

Directed recombination at the *rpoB* allele has been documented for *R*. *prowazekii* and *R*. *typhi*, generating rifampicin-resistant or GFP-expressing recombinants, respectively (Rachek et al., 1998; Troyer et al., 1999). Recombination at the *gltA* locus leading to insertion of an *ereB* erythromycin resistance marker has also been documented (Rachek et al., 2000). Gene knockout via allelic exchange has only been reported for the *pld* gene in *R. prowazekii*, which was replaced by the *arr-2* marker (Driskell et al., 2009). No other allelic exchange examples have been published since the *pld* knockout in 2009, suggesting that technical challenges exist that have not been overcome. Targeted gene inactivation using a group II intron was reported for the *ompA* gene in *R. rickettsii* using *rpsl^P^* to drive the expression of the intron and resistance marker *arr-2* (Noriea et al., 2015). The *ompB^P^* and *gltA^P^* promoters were also tested for GII intron expression and resulted in mutant production but yielded fewer mutants per transformation than the *rpsl^P^* construct. Lastly, targeted gene knockdown has been achieved in *R. typhi* (*ompB*) and *R. montanensis* (*rickA*) using peptide nucleic acids (PNAs) (Pelc et al., 2015). PNAs were introduced into bacteria via electroporation, and bacteria were then used to infect L929 or Vero cells without selection following a 1-h recovery period. PNA-treated *R. montanensis* were also used to infect *Dermacentor variabilis* ticks. PNA-treated bacteria exhibited moderate reduction in production of the targeted proteins and showed reduced infectivity, suggesting that PNAs could be useful for targeted silencing of essential genes. However, as selection is not employed with PNAs, the phenotypes measured are at the population level and would reflect protein production from both transformed and untransformed bacteria.

#### Shuttle vectors and regulated gene expression

Rickettsial plasmids were first identified in the transitional group member *R. felis* (Ogata et al., 2005) and have since been found in multiple SFG species varying from 12 to 83 kbp, which suggests an upper size limit of ~80 kbp for vectors (El Karkouri et al., 2016). The primary Rickettsial backbone for *E*. *coli*–*Rickettsia* spp. shuttle vectors is derived from the pRAM18 plasmid found in *R. amblyommatis* (SFG), which also carries two other plasmids, pRAM23 and pRAM32 (Baldridge et al., 2010a; Burkhardt et al., 2011). Modifications to pRAM18 included the addition of *arr-2* and *gfp*_UV_ allowing for selection of transformants with rifampicin and screening via GFP expression along with the insertion of a multiple-cloning site yielding pRAM18dRGA and pRAM18dRGA(MCS), respectively. A number of accessory plasmid genes were removed from pRAM18 to reduce its size, leaving the *parA* and *dnaA* genes from the native plasmid intact to support replication in *Rickettsia* spp. The *E. coli* pGEM backbone with the *bla* marker was inserted to allow for replication and ampicillin selection in *E. coli*. The copy number of pRAM18dRGA varies across species from ~1 in *R. prowazekii* (Wood et al., 2012) to 13 in *R. bellii* (Burkhardt et al., 2011). Plasmid stability in the absence of selection has been observed for *R. conorii* in a mouse infection model up to 5 days postinfection (Riley et al., 2016). However, Hauptmann et al. found that the plasmid was lost from *R*. *typhi* after ~3 weeks in the absence of rifampicin (Hauptmann et al., 2017). The pRAM18dRGA base vector has also been used to transform the non-pathogenic tick endosymbionts *R*. *buchneri* and *R*. *peacockii* using either rifampicin or spectinomycin [pRAM18dSGA, (Oliver et al., 2014)] selection, respectively (Kurtti et al., 2016).

While a number of foreign and native promoters have been used to express genes in *Rickettsia* from either plasmid or chromosomal locations, the use of inducible promoters has yet to be reported. Inducible expression systems used in *Coxiella* and *Chlamydia* would appear to be applicable for *Rickettsia* spp.

## Current limitations and future directions

Genetic transformation of many obligate intracellular bacteria, once thought genetically intractable, has been achieved. The most common technique to introduce DNA into bacterial cells is electroporation and is frequently used with the Himar1 transposon system to create random insertional mutants and fluorescent strains and to complement natural mutants. Two additional methods of DNA uptake have been established. First, the use of a more traditional CaCl_2_-based chemical competence system has been developed for *Chlamydia*, this together with *Chlamydia*’s natural ability to swap gDNA between strains during infection suggests an inherent natural competence. The second method is the use of polyamidoamine dendrimers to transport DNA into either purified bacteria or into bacteria infecting host cells. This technique has been shown to work for both *Chlamydia* and *Anaplasma* and if further developed could provide an easy way to transform obligate intracellular bacteria without the need to first purify the bacteria. Antibiotic resistance continues to be the mainstay for positive selection of transformants but is hampered by restrictions due to clinical use, background resistance, and the number of individual resistance cassettes per bacterium (i.e., *C. burnetii* is limited to using only *cat*, *bla*, and *aphA*). The recent development of antibiotic-free selection has allowed the expansion of potential selection methods and includes the use of the bialaphos herbicide resistance gene *bar* in *Anaplasma* and the genetic complementation of natural amino acid auxotrophies to lysine, arginine, proline, and tyrosine in *C. burnetii*. The use of a Cre–*lox* system would permit removal of selectable markers allowing for their reuse in a second round of transformation in an already genetically modified genome.

Although there is widespread use of transposons in obligate intracellular bacteria to create random mutants, hoping insertions land within a specific gene(s) of interest requires either luck and/or screening large numbers of transformants, assuming the gene is not essential. Homologous recombination has been observed in a few obligate intracellular bacteria, but its general use to create specific mutants by allelic exchange has not become mainstream, even in *C. burnetii* where this process is aided by axenic media. For many obligate intracellular bacteria, this can be due to low transformation efficiency, poor antibiotic selection, low recombination efficiency, and/or selection of targets essential for intracellular growth. The development of nutritional-based selection markers in *C. burnetii* has made the generation of site-directed mutants more efficient. In lieu of a viable homologous recombination system, the use of the group II mobile intron retargeting system may be possible as this technology relies on site-specific insertion of a group II intron into the gene of interest. Targeted knockdown of specific gene expression is another avenue that can be exploited to examine the function of encoded proteins. This process can be achieved in two different ways. First, the use of PNA molecules to inhibit short-term mRNA translation, as used in *Rickettsia*, and second, the use of CRISPR interference that was recently developed for *Chlamydia* to prevent transcription initiation or transcription of specific gene targets. The CRISPRi system has the added benefit of being an inducible system. Recently, a miniTn7 version of a CRISPRi system has been developed for *C. burnetii* that allows specific targeting of genes via a chromosomally inserted CRISPRi module, including those essential for axenic growth and/or growth in host cells (Wachter et al., 2022). Complementation of CRISPRi knockdowns was achieved by co-expressing copies of the transcriptionally silenced gene whereby the CRISPRi-targeted region was codon optimized to prevent knockdown of the complementing transcript.

Complementation of transposon and site-directed mutants is important for fulfillment of molecular Koch’s postulates. Three of the species described here, *Chlamydia*, *C. burnetii*, and *Rickettsia*, have been modified to satisfy the postulates using multicopy shuttle vectors, and whereas in most cases this works well, having multiple copies of the complementing gene may cause unexpected results. For *E. chaffeensis*, molecular Koch’s postulates were achieved using allelic exchange of a linear PCR product containing an alternate antibiotic selection and a full-length copy of the previously mutated gene. The widespread use of the miniTn7 transposon system to complement mutants with a single wild-type copy of the disrupted gene containing its native promoter has been demonstrated for *C. burnetii*. The transposase from this two-plasmid transposition system recognizes a 30-bp sequence normally found in the end of the *glmS* gene. An analysis of the genomes from the different species described here indicated that this system can likely be used in *Chlamydia* and some *Anaplasma* species (**Figure 4**). This utility can easily be expanded to all species by adding the 30-bp recognition sequence to the DNA used to create the mutants (i.e., added to the Himar1 transposon). Two important aspects need attention when using systems developed in one bacterium for another: 1) Making sure the codon usage is similar to that of the recipient species. *Coxiella*, *Chlamydia*, and *Anaplasma* all have a GC content (~42%) similar to *E*. *coli*, whereas *Rickettsia*, *Ehrlichia*, and *Orientia* have a GC content of ~30%; therefore, most systems that work in *E*. *coli* will likely work in the former species but not the latter, although specific codon biases will still need examining (https://www.kazusa.or.jp/codon/ provides codon usage for most species). 2) The use of species-specific or inducible promoters to drive gene expression of system genes (i.e., transposases, antibiotic selection, reporters). Here, the choice of promoter strength and/or activity (i.e., early vs late in the development cycle or constitutive promoters; host infection model [mammal versus tick]) is important, and in the case of inducible promoters, complete redesigns may be warranted if the promoter does not work in the targeted bacterium (i.e., adding LacI binding sites to a species-specific promoter).

The lack of methods to genetically manipulate obligate intracellular bacteria has in the past hampered the ability to study the requirements needed for survival and virulence of these organisms. Due to the persistence and hard work of the researchers described here, significant achievements have been made enabling genetic modification of many of the bacteria in this field. New genetic technologies are still needed (e.g., universal CRISPRi gene silencing) in order to study these bacteria, but adaptions of older methods will likely also play a role (e.g., the use of the traditional CaCl_2_ method for making bacteria competent for DNA uptake used for *Chlamydia* or chemical mutagenesis as used for *Chlamydia*). The development of an axenic medium has undoubtedly changed the nature of genetic manipulation for *C. burnetii*. Axenic media not only provide an outlet for studying genes essential for intracellular growth but also allow the development of nutritional-based selection systems, expanding the number of markers able to be used for genetic transformation. The development of an axenic medium for *Chlamydia*, *Anaplasma*, and *Ehrlichia* has begun, and while it is a daunting task, it is worth the risk. Preliminary data have shown robust extracellular protein and DNA biosynthesis for all species indicating activation of metabolic activity and give hope for an axenic medium that supports robust growth in the future. With almost one-third to more than one-half of all the publications for each species described here coming in the last 10 years, it indicates the importance of these (re)emerging pathogens. This is evident in the increased enthusiasm toward the development of genetic tools. Through genetics, we can gain an understanding of these organisms’ lifestyles and interactions with their hosts and help identify important virulence factors that may aid in the development of new or novel treatments and vaccines.

## Author contributions

DF and PB devised, wrote, and edited the review. All authors contributed to the article and approved the submitted version.
